# EZH2 and intracellular Ca^2+^ signals interdependently coordinate alloreactive and CAR-T-cell responses

**DOI:** 10.1038/s41423-026-01413-y

**Published:** 2026-04-22

**Authors:** Ying Wang, Qingrong Huang, Yan Zhou, Robert Hooper, Ruqayyah Sanders-Braggs, Mimi Chen, Yuanyuan Tian, Tatiana Kent, Richard Pomerantz, Gennaro Clando, Woonbok Chung, Jean-Pierre J. Issa, Jonathan Soboloff, Yi Zhang

**Affiliations:** 1 Center for Discovery and Innovation, Hackensack University Medical Center, Hackensack Meridian Health, Nutley, NJ USA; 2 Clinical Immunology Core, Center for Discovery and Innovation, Hackensack University Medical Center, Nutley, NJ USA; 3 Biostatistics and Bioinformatics Facility, Fox Chase Cancer Center, Temple University, Philadelphia, PA USA; 4 Fels Institute and Department of Cancer Cellular Biology, Lewis Katz School of Medicine, Temple University, Philadelphia, PA USA; 5 Department of Biochemistry and Molecular Biology, Thomas Jefferson University, Sidney Kimmel Cancer Center, Philadelphia, PA USA; 6 Coriell Institute for Medical Research and Cooper Medical School at Rowan University, Camden, NJ USA; 7 Genetic, Environmental and Inhalational Disease, University of Kansas Medical Center, Kansas City, MO USA

**Keywords:** EZH2, ER calcium, Allogeneic HSCT, T cell, GVHD, Epigenetic regulation, Immunology, Bone marrow transplantation

## Abstract

In graft-versus-host disease (GVHD), Ca^2+^ signals in alloreactive T cells are carefully controlled to determine whether cells survive or thrive, although how this is accomplished during GVHD remains poorly defined. We demonstrate that EZH2, a chromatin-modifying enzyme, promotes alloreactive T-cell survival in GVHD by acting as a Ca^2+^ signaling brake to limit excessive intracellular Ca^2+^ responses. *Ezh2* loss led to the upregulation of gene programs that promote effector differentiation in activated T cells, coincident with enhanced intracellular Ca^2+^ responses that ultimately caused massive cell death. Conditional deletion of *Stim1* (required for cytosolic Ca^2+^ entry) led to “synthetic rescue” of *Ezh2*-null T cells by protecting them from cell death without interfering with effector differentiation, resulting in severe GVHD. Interestingly, *Stim1* expression was unaffected by EZH2, whereas the expression of the endoplasmic reticulum Ca^2+^ release channel inositol 1,4,5-trisphosphate receptor 2 (*Itpr2*) was suppressed by EZH2. Notably, EZH2 and Ca^2+^ signals served mutually opposing roles in controlling the expression of genes in chimeric antigen receptor (CAR) T cells. Inhibiting Ca²⁺ signaling restored EZH2 function in CAR-T cells, significantly improving their antitumor activity. Our findings reveal the interdependent roles of EZH2 and Ca^2+^ signals in coordinating antigen-activated T-cell responses that mediate alloimmunity and tumor immunity.

## Introduction

Graft-versus-host disease (GVHD) is caused by donor T cells that react to differences in histocompatibility between the host and the donor after allogeneic hematopoietic stem cell transplantation (allo-HSCT). It is initiated by antigen-presenting cell priming of donor T cells, followed by robust proliferation and differentiation of alloreactive T cells that mediate tissue injury, primarily in the skin, liver and gut [1–3]. Ca^2+^ signaling plays fundamental roles in the proliferation, effector differentiation and expansion of antigen-activated T cells [4–7]. Calcineurin inhibitors (CNIs), which target the Ca^2+^ signaling-activated NFAT/IL-2 pathway, constitute the standard GVHD prophylactic regimen. However, when this approach is used, GVHD still occurs, albeit with a substantial delay [1–3]. Recent studies suggest that CNI treatment induces the formation of alloreactive memory and progenitor effector T cells that have enhanced survival and self-renewal capacity [8, 9], indicating that inhibition of Ca^2+^ signaling favors the persistence of alloreactive T cells. Alternatively, the enhanced intracellular Ca^2+^ response observed after ablation of inositol 1,4,5-trisphosphate 3-kinase B, a negative regulator of intracellular Ca^2+^ release, in donor T cells induces their apoptosis and the subsequent reduction in GVHD [10]. Thus, delicately controlling Ca^2+^ responses is crucial for inducing productive alloreactive T-cell responses. However, the epigenetic mechanisms that regulate balanced intracellular Ca^2+^ responses in activated T cells have not yet been defined.

EZH2, a chromatin-modifying epigenetic regulator, catalyzes the trimethylation of histone H3 at lysine 27 (H3K27me3) and plays essential roles in multiple cell processes, including cell proliferation, survival, and differentiation [11–17]. EZH2 also plays a central role in T cells, regulating responses to infection, tumors, and alloantigens [18–22]. It restrains the terminal differentiation of effector CD8^+^ T cells, promotes the formation of memory CD8^+^ T cells, regulates the lineage specification and identity maintenance of helper CD4^+^ T cells, and prevents the death of antigen-activated T cells. However, the loss of EZH2 in T cells results in the inhibition of GVHD and antitumor activity, largely due to antigen-activated T-cell death [18–20, 23], which has limited our efforts to elucidate how EZH2 regulates T-cell immunity.

Roles for Ca^2+^ signaling in activated T cells have been established in cell death and dysfunction [4, 6, 24]. Antigen binding to the T-cell receptor (TCR) activates phospholipase C (PLC), which converts phosphatidylinositol 4,5-bisphosphate (PIP_2_) to inositol 1,4,5-trisphosphate (InsP_3_). Diffusible InsP_3_ binds the InsP_3_ receptor (InsP_3_R) on the endoplasmic reticulum (ER) to trigger Ca^2+^ release [4, 25–27]. The resulting ER Ca^2+^ depletion is the trigger for Stromal Interaction Molecule 1 (STIM1) activation, leading to ORAI-mediated Ca^2+^ entry (which is termed store-operated calcium entry, or SOCE) [4–6, 24]. Increased intracellular Ca^2+^ accumulation may trigger T-cell death through multiple mechanisms. Ca^2+^ signaling increases FAS ligand and TRAIL expression and activates intrinsic apoptosis primarily by upregulating the expression of proapoptotic genes [4, 28]. They may also facilitate Ca^2+^-dependent cytochrome-C release from mitochondria to induce cell death [24, 29]. Deletion of *Stim1* or *Orai1* in T cells leads to a reduction in Ca^2+^ levels and resistance to cell death [24, 28]. Given the importance of EZH2 in converting antigenic stimuli to gene programs that regulate antigen-driven T-cell responses [4, 6], we hypothesized that EZH2 and Ca^2+^ signaling could cooperate to control T-cell immune responses.

Using both experimental GVHD and chimeric antigen receptor (CAR) T-cell therapy models, we demonstrate that EZH2 acts as a Ca^2+^ signaling brake in activated T cells, protecting them from heightened Ca^2+^ response-induced cell death. On the other hand, Ca^2+^ signaling can modulate EZH2 function in CAR-T cells. The inhibition of Ca^2+^ signaling enhanced EZH2 function in cultured CAR-T cells, improving their efficacy in tumor control in vivo following infusion.

## Results

### EZH2 acts as a brake for the generation of intracellular Ca^2+^ signals

**Fig. 1:**
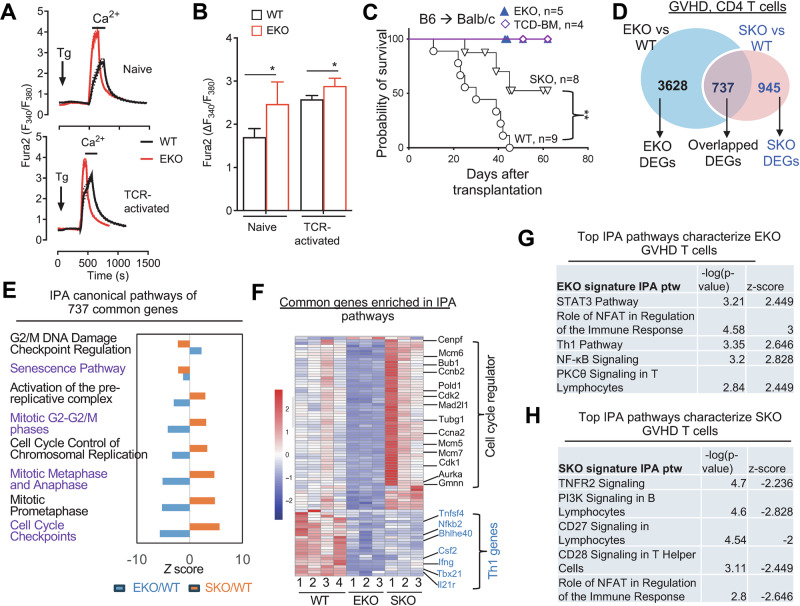
EZH2 acts as a brake for the generation of intracellular Ca^2+^ signals. **A**, **B** WT and EKO CD8^+^ T_N_ cells were activated with anti-CD3/CD28 antibody for 3 days. Both CD8^+^ T_N_ cells and TCR-activated and CD8^+^ cells were loaded with fura-2 for 1 h and depleted ER Ca^2+^ stores with Thapsigargin (Tg, 2 μM) and monitored under a fluorescent microscope for Ca^2+^ entry (SOCE). Each trace (black: WT; red: EKO) represents the average ratio of F_340/380_ of all recorded cells in the imaging field. Data shown are representative of 3 independent experiments. Two-way ANOVA followed by post-hoc tests was performed. *p < 0.05. Data from a representative of three experiments. **C** Balb/c mice were given total body irradiation (TBI, 4.5 Gy on day -1 and 4 Gy on day 0) followed by infusion of 5 × 10^6^ B6/SJL mouse (CD45.1^+^CD45.2^-^) T cell-depleted bone marrow (TCD-BM) alone or together with B6 (CD45.1^-^CD45.2^+^) naive WT, EKO T cells, or SKO T cells (5 × 10^5^ CD4^+^ and 2 × 10^5^ CD8^+^ T cells). The graph shows the survival of Balb/c recipients. Statistical analysis was performed with the Log-rank (Mantel–Cox) test. **D**–**H** Two weeks after allo-HSCT, donor CD4^+^ T cells were FACS-purified from Balb/c recipients for performing RNA-seq analysis. **D** Venn diagram shows the number of DEGs comparing EKO versus WT to SKO versus WT T cells. **E** Z scores of enriched pathways were shown, which were performed on the 737 overlapped DEGs. These pathways were regulated differently by EZH2 and STIM1. **F** Heatmap shows the expression profile of the subset of the common genes regulating cell cycle and Th1 differentiation. **G** Table shows enriched top pathways analyzed with DEGs comparing EKO versus WT. **H** Table shows enriched top pathways analyzed with DEGs comparing SKO versus WT

To examine the association between EZH2-regulated gene programs and Ca^2+^ signaling in activated T cells, we first used Ingenuity Pathway Analysis (IPA) to reanalyze RNA-sequencing (RNA-seq) data from our previous studies [18]. The loss of *Ezh2* (EKO) in TCR-activated CD8^+^ T cells led to significantly increased expression of genes associated with regulators involved in the activation of Ca^2+^ signaling, including genes encoding NFAT, IL-2, and CNI (Fig. S1a). To assess whether *Ezh2* loss affects intracellular Ca^2+^ levels in T cells, we used the fluorescent probe Fura-2 to measure cytosolic Ca^2+^ levels in naïve (T_N_) and TCR-activated CD8^+^ T cells as previously described [30]. Following thapsigargin-induced ER Ca^2+^ depletion, EKO CD8^+^ T cells exhibited significantly enhanced Ca^2+^ influx mediated by SOCE (Fig. 1A, B). Since *Ezh2*-null T cells maintain their ability to proliferate upon TCR activation [18, 20], increased cytosolic Ca^2+^ entry in *Ezh2*-null T cells was unlikely the result of aberrant T-cell activation. This effect was not associated with increases in EZH1 or other components of PRC2 (EED and SUZ12) (Fig. S1b).

We next examined whether the enhanced SOCE in EKO T cells is associated with alterations in STIM1, the principal ER Ca^2+^ sensor required for SOCE activation. Although *Stim1* expression did not increase in EKO CD8^+^ T cells (Fig. S1c), the STIM1 protein exhibited a polarized distribution in inactivated naïve CD8^+^ T cells (Fig. S1d). Because STIM1 polarization reflects ER Ca^2+^ release, which subsequently triggers SOCE [4–6, 24], this poise-to-activation status suggests that increased Ca^2+^ influx is driven by enhanced STIM1-mediated SOCE in EKO T cells.

Because STIM1-mediated Ca^2+^ signaling activates key transcription factors (TFs) such as NFAT [4, 24], we next investigated whether EZH2-dependent transcriptional programs converge with STIM1-mediated Ca^2+^ signaling to regulate antigen-driven T-cell responses. In line with *Stim1* loss causes severe immunodeficiency [31], C57/BL6 (B6) T cells with conditional *Stim1* knockout (SKO) induced significantly less severe GVHD than WT T cells, with 62.5% of the SKO T-cell recipients surviving; while conditional *Ezh2* knockout B6 T cells failed to induce the disease (Fig. 1C), consistent with our previous observations [19, 20, 32] and others [33].

To determine whether the two pathways share common downstream gene programs, we performed transcriptomic profiling using FACS-purified alloreactive CD4^+^ T cells from the spleen and liver of GVHD mice at the effector phase (14 days after T-cell infusion). Compared with their WT counterparts, CD4^+^ EKO T cells had 4365 differentially expressed genes (DEGs), whereas 1682 DEGs were identified in CD4^+^ SKO T cells. Among these SKO DEGs, 737 genes (43.8%, *p* value < 2.0e-16; Fisher’s exact test) overlapped with those found in EKO T cells (Fig. 1D). These overlapping DEGs were enriched in molecular pathways associated with cell cycle checkpoints, mitotic prometaphase, the mitotic G2–G2/M phase and the senescence pathway (Fig. 1E, F). The loss of *Ezh2* led to the inhibition of gene programs that regulate cell proliferation and DNA replication, whereas the deletion of *Stim1* resulted in the activation of these gene programs (Fig. 1E, F).

In IPA, z scores greater than or equal to 2.0 indicate significant activation, whereas z scores less than or equal to -2.0 indicate significant inhibition [34]. Further characterization revealed that the DEGs in CD4^+^ EKO T cells were associated with activation of the Th1 pathway, NFAT regulation of the immune response and NFκB signaling (Fig. 1G). In contrast, DEGs associated with the inhibition of PI3K signaling, NFAT regulation of immune responses, TNFR2 signaling and CD28 signaling in T helper cells were detected in CD4 ^+^ SKO T cells (Fig. 1H). Thus, EZH2-regulated gene programs and STIM1-mediated Ca^2+^ signaling play opposing roles in alloreactive T cells. Furthermore, a large proportion of those Ca^2+^ signal-activated genes are repressed by EZH2, indicating that EZH2 may act as a brake on intracellular Ca^2+^ signaling.

### Blockade of STIM1-mediated Ca^2+^ signaling rescues nonviable *Ezh2*-null GVHD T cells

**Fig. 2:**
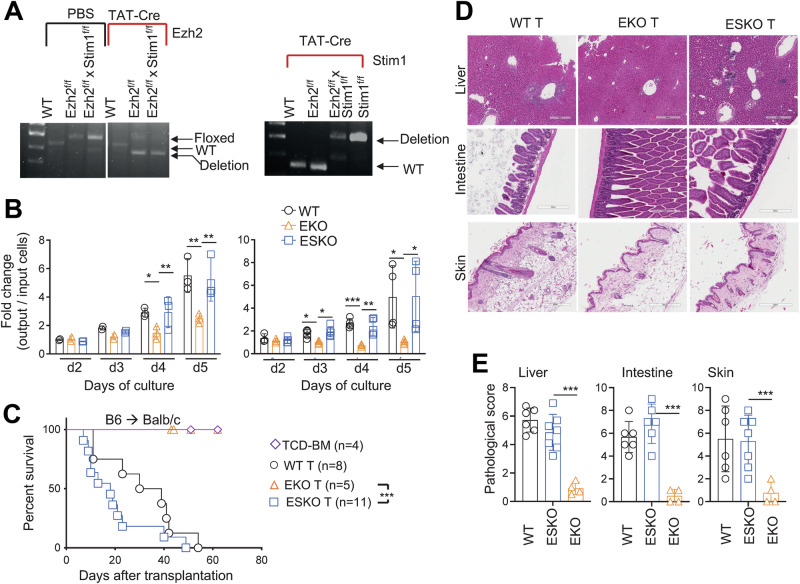
Blockade of STIM1-mediated Ca^2+^ signals rescues the GVHD-inducing capacity of Ezh2-null T cells. **A** Gel images show the deletion efficiency of *Ezh2* and *Stim1* by TAT-Cre treatment in inactivated T cells maintained under homeostasis conditions. **B** Bar graphs show the recovery rate of in vitro cultured WT, EKO, ESKO CD4^+^ and CD8^+^ T cells on day 2, 3, 4, 5 post-activation with anti-CD3/CD28 antibodies. Statistical analysis was performed on at least 3 biological replicates. Representative data from three independent experiments are shown. **C** Curve shows the survival of balb/c recipients infused with TCD-BM (5 × 10^6^) and WT, EKO or ESKO CD4^+^ and CD8^+^ T cells (5 × 10^5^ CD4^+^ + 2 × 10^5^ CD8^+^). **D** Representative H&E staining images of liver, intestine and skin collected from balb/c recipients infused with TCD-BM and the indicated genetically manipulated T cells 3-10 weeks post-HSCT. **E** Bar graphs show the pathological scores of GVHD organs shown in (**D**). Student’s *t* test was used for two-group comparison. One-way ANOVA followed by post-hoc tests was performed for pairwise comparisons among multiple groups. Statistical analysis on mice survival was performed with the Log-rank (Mantel–Cox) test. *p < 0.05, **p < 0.01, ***p < 0.001

To test whether excessive STIM1-mediated Ca^2+^ signaling contributes causally to the loss of viability in EKO effector T cells, we examined whether deletion of *Stim1* could rescue the functional defects of EKO T cells in inducing GVHD. Rescue of EKO T-cell viability by *Stim1* deletion would provide direct genetic evidence that EZH2 protects against alloreactive T-cell death by suppressing STIM1 activity. We first assessed the effect of *Stim1* deletion on the GVHD-inducing capacity of allogeneic EKO T cells. Cre recombinase fused with the TAT peptide (TAT-Cre) catalyzes site-specific recombination between two loxP DNA sites [35]. By delivering TAT-Cre to *Ezh2*^fl/fl^. *Stim1*^fl/fl^ B6 T cells cultured in the presence of IL-2 + IL-7 + IL-15, we deleted *Stim1* and *Ezh2* to generate *Ezh2*^–/–^.*Stim1*^–/–^ T cells (named ESKO, Fig. S2a). Consistent with earlier observations (Fig. 1A), the deletion of *Ezh2* increased the SOCE-dependent Ca^2+^ oscillation amplitude (Fig. S2b). Although ESKO T cells lacked Ca^2+^ oscillations (Fig. S2b), their in vitro expansion remained comparable to that of WT cells, in contrast to EKO cells, whose expansion was markedly impaired (Fig. 2B). Moreover, introducing either ESKO T cells (*n* = 11) or WT T cells (*n* = 8) to BALB/c mice led to severe GVHD, as manifested by survival and significant inflammation in the liver, intestine and skin through histological examination, whereas EKO T-cell recipients (*n* = 5) failed to develop GVHD (Fig. 2B–D). These results reveal that the conditional deletion of *Stim1* leads to the “synthetic rescue” of *Ezh2*-null T cells by protecting them from cell death.

**Fig. 3:**
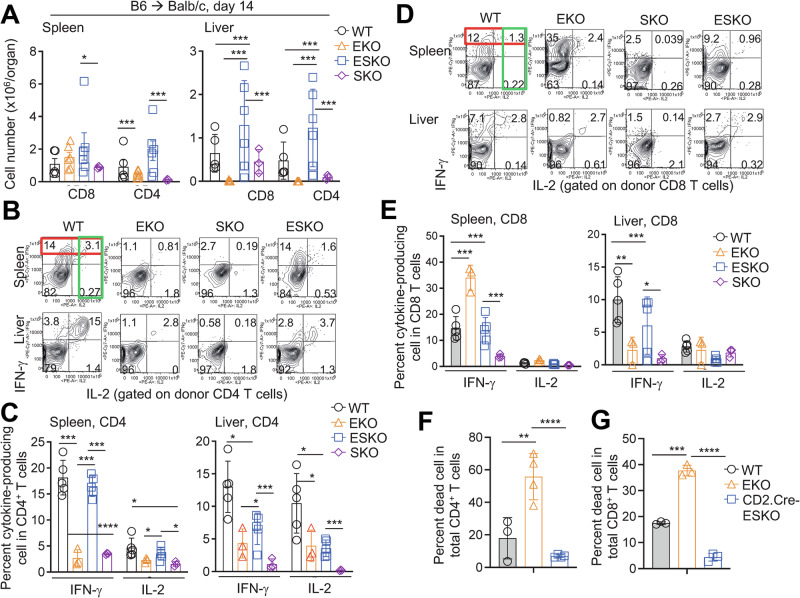
Blockade of STIM1-mediated Ca^2+^ signals rescues non-viable *Ezh2*-null GVHD T cells. **A** Graphs show the numbers of donor CD8^+^ and CD4^+^ T cells recovered from the spleens and livers of balb/c recipients 2 weeks post-HSCT. **B**, **C** Flow plots and bar graphs show the cytokine-producing capacity (IFN-γ- and IL-2) of donor CD4^+^ T cells isolated from the spleens and livers of balb/c recipients 2 weeks post-HSCT. **D**, **E** Flow plots and bar graphs show the cytokine-producing capacity (IFN-γ- and IL-2) of donor CD8^+^ T cells isolated from the spleens and livers of Balb/c recipients 2 weeks post-HSCT. Bar graphs show the dead cell frequency in cultured Th1 (**F**) and CD8^+^ (**G**) T cells. Statistical analysis was performed on at least three independent experiments. One-way ANOVA followed by post-hoc tests was performed for pairwise comparisons among multiple groups. *p < 0.05, ***p < 0.001, ****p < 0.0001

To elucidate the mechanism by which conditionally deleting *Stim1* in EKO T cells restored their ability to mediate GVHD, we isolated donor T cells from the spleen and liver of BALB/c mice that received WT, EKO, SKO and ESKO T cells (CD4^+^ and CD8^+^) 14 days after transplantation. Compared with WT T cells, both EKO and SKO T cells showed impaired in vivo expansion in the liver, whereas combined deletion of *Ezh2* and *Stim1* in donor T cells rescued their proliferation and expansion during GVHD (Fig. 3A). Furthermore, compared with EKO and SKO T cells, alloreactive ESKO and WT CD4^+^ T cells produced  higher frequencies of IFN-γ- and IL-2-producing effector T cells in the liver (Fig. 3B, C). In alloreactive CD8^+^ T cells, the deletion of *Ezh2* and/or *Stim1* did not significantly affect the production of IL-2 (Fig. 3D, E). However, compared with SKO CD8^+^ T cells, ESKO CD8^+^ T cells generated approximately 3- and 5-fold higher frequencies of IFN-γ-producing effector cells in the spleen and liver, respectively (Fig. 3D, E). These findings suggest that the deletion of both *Ezh2* and *Stim1* functionally rescued the expansion and cytokine production of alloreactive T cells lacking either gene alone, thereby defining the EZH2-STIM1 axis that regulates Ca^2+^ signaling intensity, which ultimately dictates antigen-driven T-cell survival during GVHD.

To exclude a model-specific effect of TAT-Cre-mediated deletion of *Ezh2* and *Stim1* in T cells, we crossed CD2-Cre B6 mice with *Ezh2*^fl/fl^.*Stim1*^fl/fl^ B6 mice to generate CD2-Cre.*Ezh2*^fl/fl^.*Stim1*^fl/fl^ B6 mice (CD2.Cre-ESKO). CD2-Cre-mediated deletion of *Ezh2* and *Stim1* occurs at the common lymphoid progenitor cell stage [36]. Upon stimulation with anti-CD3/CD28 antibodies, CD2.Cre-ESKO T cells had a 6- to 7-fold lower frequency of dead cells 4 days after activation compared to EKO T cells (Figs. 3F, G and S3b). Transplantation of B6 CD2.Cre-ESKO T cells (*n* = 11) induced lethal GVHD in 36.4% of BALB/c recipients (Fig. S3a), indicating that the capacity to mediate GVHD was partially restored. We recognize that this is a lower efficiency than that observed with the TAT. Cre-ESKO T cells. The frequency of double positive cells significantly decreased in the CD2.Cre-ESKO cells, reflecting abnormal thymic development (Fig. S3c, d), which may account for this discrepancy. Nevertheless, these data provide qualitative support for our conclusion that increased death in EKO T cells is caused by STIM1-dependent Ca^2+^ signaling.

### EZH2- and STIM1-mediated Ca^2+^ signaling interdependently regulates T-cell responses

**Fig. 4:**
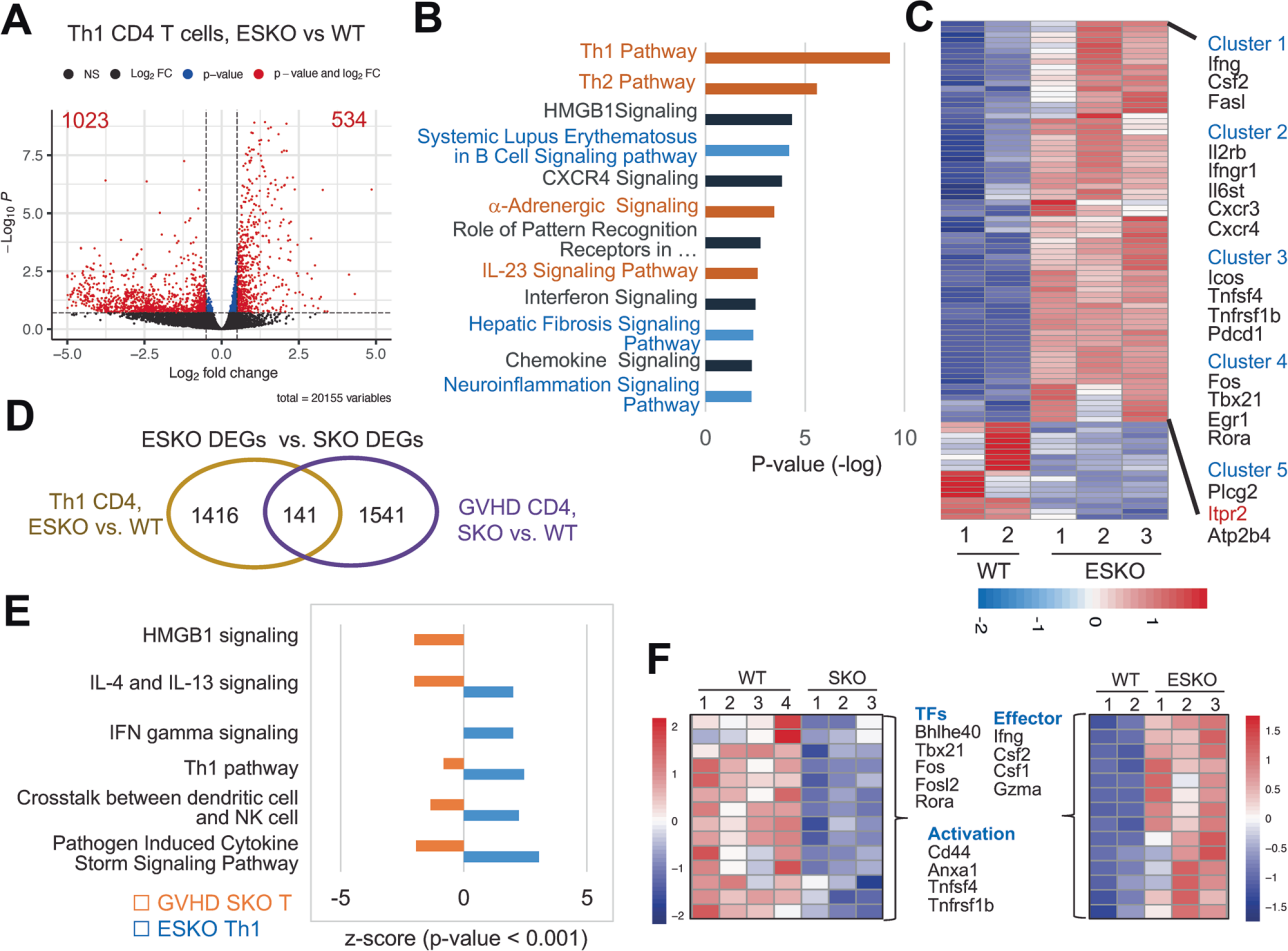
EZH2 and STIM1-mediated Ca^2+^ signals interdependently regulate T cell responses. RNA-seq was performed on cultured WT and ESKO Th1 cells. **A** Volcano plot shows DEGs comparing ESKO vs WT with a cutoff of FDR < 0.2 and fold change >1.5. **B** IPA canonical pathway analysis of DEGs between ESKO and WT Th1 cells. **C** Heatmap shows DEGs with higher IPA enrichment scores shown in (**B**). Blocks are annotated by their functions. **D** Venn diagram shows the numbers of DEGs comparing cultured ESKO vs WT Th1 cells overlapped with DEGs comparing SKO vs WT GVHD CD4^+^ T cells. **E** IPA canonical pathway analysis of the overlapped 141 DEGs coregulated by EZH2 and STIM1. **F** Heatmap of log ratios shows 21 DEGs identified based on IPA canonical pathways with reversely correlated *z*-scores

To define the gene programs controlled by the interplay between EZH2 and STIM1-dependent Ca^2+^ signaling, we performed RNA-seq analysis of WT and ESKO Th1 CD4^+^ T cells generated under Th1 culture conditions. Compared with WT Th1 cells, ESKO Th1 cells upregulated 534 genes and downregulated 1023 genes (Fig. 4A). These DEGs were associated with Th1 and Th2 responses, chemokine signaling, IFN signaling and inflammatory disease signaling pathways (lupus, hepatic fibrosis and neuroinflammation) (Fig. 4B). These DEGs can be broadly classified into five major clusters: (i) effector molecules (*Csf2, Ifng, and Fasl*); (ii) cytokine and chemokine receptors (*Il2rb, Ifngr1, Il6st, Cxcr3, and Cxcr4*); (iii) costimulatory and inhibitory receptors (*Icos and Pdcd1*); (iv) transcription factors critical for effector differentiation (*Tbx21 and Fos*); and (v) regulators of Ca^2+^ signaling and homeostasis (*Plcg2, Iptr2, and Atp2b4*) (Fig. 4C). Notably, although STIM1-mediated Ca^2+^ signaling was critical for the induction of IFN-γ-producing Th1 cells [37], the deletion of *Ezh2* rescued the ability of the SKO T cells to produce effector cytokines (Fig. 4C). Thus, EZH2- and STIM1-mediated Ca^2+^ signaling play mutually opposing roles in controlling the expression of functional genes in activated T cells, allowing them to interdependently coordinate T-cell proliferation, differentiation and survival during immune response.

To elucidate the key molecular pathways through which EZH2 represses Ca^2+^ signaling-regulated gene programs, we compared the DEGs identified in ESKO Th1 cells (Fig. 4A–C) to those detected in alloreactive SKO T cells isolated from mice with GVHD (Fig. 1C–E). We identified 141 DEGs that overlapped between ESKO Th1 cells and SKO T cells from mice with GVHD (Fig. 4D). The absence of *Stim1* alone resulted in decreased expression of genes associated with IL-4 and IL-13 signaling, Th1 pathway, and pathogen-induced cytokine storm signaling (Fig. 4E). However, deletion of *Ezh2* from *Stim1*-KO Th1 cells (i.e., ESKO Th1 cells) activated these repressed gene programs (Fig. 4E). These inversely correlated representative DEGs (FC > 1.5, *p* < 0.05) included (Fig. 4F) effector molecules (*Ifng, Csf2, Csf1, and Gzma*), activation markers (*Tnfsf4, Tnfrsf1b, CD44, and Anxa1*), and transcription factors (*Tbx21, Bhlhe40, Rora and Fos*, which promote Th1 differentiation and effector cell proliferation). Thus, EZH2 restrains a subset of Ca^2+^ signaling-responsive gene programs that promote effector T-cell development and survival.

### EZH2 represses T-cell expression of the intracellular Ca^2+^ release channel InsP_3_R2

**Fig. 5:**
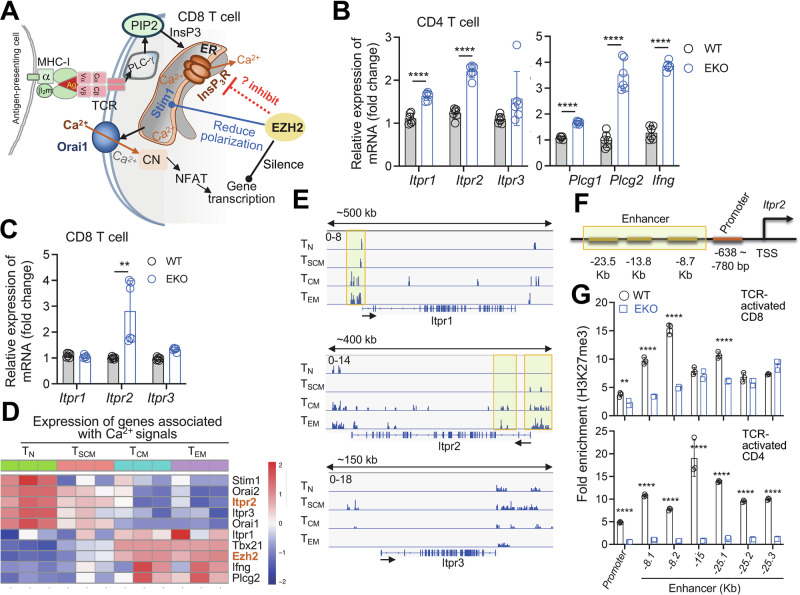
EZH2 represses T cell expression of intracellular Ca^2+^ release channel InsP_3_R2. **A** Diagram depicts how TCR activation triggers the formation of the CRAC channel, the role of InsP3R and the signaling pathway. **B** Graphs show the expression of genes encoding calcium channels in 3-day cultured EKO and WT CD4^+^ T cells. **C** Graph shows the expression of *Itpr1*, *Itpr 2* and *Itpr3* in 3-day cultured EKO and WT CD8^+^ T cells. **D** In-silico analysis was performed on the microarray data (series GSE67825). Heatmap shows differential expression of genes associated with Ca^2+^ signals comparing differentiated CD8^+^ effector memory T (T_EM_) cells with other subsets. **E** In-silico analysis was performed on the ChIP-seq data set (series GSE67881). Tracks show the localization of H3K27me3 in the *Itpr1*, *Itpr2* and *Itpr3* locus in T_N,_ T_SCM_, T_CM_ and T_EM_. **F** Location of CUT&RUN qPCR primers along the mouse *Itpr2* promoter and enhancer regions. **G** Bar graphs show the enrichment of H3K27me3 in the regulatory regions of *Itpr2*, indicated in (**F**), measured by qPCR. Statistical analysis was performed on at least three independent experiments. Student’s *t* test was used for two-group comparison. **p < 0.01, ****p < 0.0001

Next, we investigated the upstream mechanism through which EZH2 regulates STIM1-mediated Ca^2+^ signaling. The poise-to-activate status of STIM1 in unstimulated EKO T cells and the unchanged *Stim1* mRNA level (Fig. S1c, d) suggest that EZH2 modulates STIM1 sensitivity. STIM1 senses ER Ca^2+^ content, the release of which occurs through InsP_3_ receptors (InsP_3_Rs, encoded by *Itpr1-3*) [38] in response to TCR ligation, which stimulates PLC-γ that hydrolyzes PIP_2_ to generate InsP_3_ (Fig. 5A) [6, 25, 31, 39]. Since the expression of *Itpr2* was upregulated in ESKO Th1 cells (Fig. 4C), we hypothesized that EZH2 represses the expression of genes that regulate ER Ca^2+^ release to tune the activation status of STIM1 (Fig. 5A). RT‒qPCR analysis revealed that TCR-activated EKO CD4^+^ T cells expressed elevated levels of *Itpr1, Itpr2, Plcg1 and Plcg2* compared to WT CD4^+^ T cells (Fig. 5B). Similarly, increased *Itpr2* expression was detected in activated EKO CD8^+^ T cells (Fig. 5C).

To determine whether EZH2 directly represses the transcription of *Itpr* genes in T cells, we retrieved publicly available microarray data (series GSE67825) and H3K27me3 Ab-based ChIP-seq datasets (series GSE67881) [40]. Upon antigen activation, CD8^+^ T_N_ cells differentiate into the least differentiated T memory stem cells (T_SCM_), followed by central memory T cells (T_CM_) and effector/effector memory T cells (T_E/EM_) [41]. Compared with CD8^+^ T_N_ cells and T_SCM_ cells, CD8^+^ T_E/EM_ cells, which upregulated *Ezh2* expression, exhibited the lowest expression of *Itpr2* (Fig. 5D). ChIP-seq analysis revealed that both T_E/EM_ and T_CM_ cells presented higher levels of the H3K27me3 marker at the promoter regions of *Itpr1* and *Itpr2* loci than T_N_ and T_SCM_ cells (Fig. 5E). Cleavage Under Targets and Release Using Nuclease (CUT&RUN) followed by qPCR analysis revealed that the loss of EZH2 resulted in a significant reduction in the presence of H3K27me3 at the promoter and enhancer regions of the *Itpr2* gene (Fig. 5F, G). We verified that the addition of tazemetostat (TAZ), which specifically reduces H3K27me3 [42], decreased cellular levels of H3K27me3 (Fig. S4a) and increased the transcription of *Itpr2* in TCR-activated T cells (Fig. S4b). Thus, EZH2 can prevent hyperactivation of Ca^2+^ signaling through repressing *Itpr2* expression in T cells.

### Inhibiting InsP_3_R2 in EKO T cells restores their ability to mediate GVHD and control leukemia

**Fig. 6:**
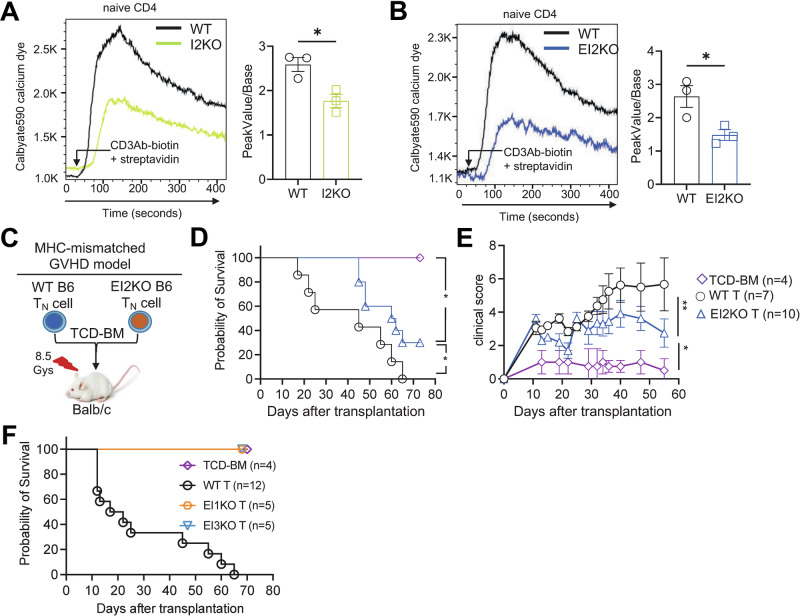
Inhibiting InsP_3_R2 in EKO T cells restores their capacity to mediate GVHD. **A** Traces show cytosolic calcium levels in I2KO and WT CD4^+^ T cells measured with Calbyate590 dye using flow cytometry. The bar graph shows the statistical analysis of the peak values represented as the mean fluorescence intensity (MFI) from at least 3 independent experiments. **B** Traces show cytosolic calcium levels in EI2KO and WT CD4^+^ T cells measured with Calbyate590 dye using flow cytometry. The bar graph shows the statistical analysis of the peak values represented as the MFI. Data shown are representative of 3 independent experiments. **C** Schematic description of Balb/c GVHD model establishment. **D** Curves show the survival. **E** clinical scores of Balb/c recipients infused with TCD-BM and WT, EKO or EI2KO CD4^+^ and CD8^+^ T cells. **F** Curves show the survival of Balb/c recipients infused with TCD-BM and WT or EI1KO or EI3KO CD4^+^ and CD8^+^ T cells. Statistical analysis was performed on at least 3 independent experiments. Student’s *t* test was used for two-group comparison. Statistical analysis on mice survival was performed with the Log-rank (Mantel–Cox) test. *p < 0.05, **p < 0.01

To test whether increased InsP_3_R2-mediated Ca^2+^ signaling in EKO T cells contributes to their impaired survival and persistence during antigen-driven immune responses, we crossed *Itpr2*^fl/fl^ B6 mice with CD4-Cre.EKO mice to generate *Ezh2*- and *Itpr2*-double knockout B6 mice (named EI2KO mice). CD4-Cre. *Itpr2*^fl/fl^ B6 mice (I2KO) were generated as controls. Deletion of *Itpr2* led to significantly decreased TCR-mediated cytosolic Ca^2+^ responses in CD4^+^ T cells (Fig. 6A), demonstrating the important contribution of InsP_3_R2 to intracellular Ca^2+^ release during T-cell activation. Similarly, decreased TCR-induced Ca^2+^ responses were also observed in EI2KO cells (Fig. 6B), suggesting the importance of InsP3R2 in mediating intracellular Ca^2+^ release. The transplantation of EI2KO T cells (*n* = 10) into lethally irradiated BALB/c mice resulted in severe acute GVHD, with 70% of them succumbing to the disease, though less severe than WT T-cell recipients (*n* = 7) (Fig. 6C–E). In contrast, deletion of *Itpr1* or *Itpr3* in EKO T cells (EI1KO, EI3KO, *n* = 5) failed to restore their ability to induce GVHD (Fig. 6F). This could be associated with the partial compensation between InsP_3_R1 and InsP_3_R3, which might form distinct InsP_3_R complex isoforms with InsP_3_R2 when the other subunit is absent [43, 44]. This hypothesis warrants further exploration in future studies. Therefore, InsP_3_R2, but not InsP_3_R1 or InsP_3_R3, plays a critical, nonredundant role in mediating death in EKO T cells. Since I2KO donor T cells caused GVHD as WT T cells did (Fig. S4c), these results suggest that pathological Ca^2+^ signaling arises specifically from unchecked *Itpr2* upregulation in the absence of EZH2, which interferes with cell survival.

To determine the impact of EZH2 on Ca^2+^ signaling in human T cells, we used the CRISPR-Cas9 method to ablate the *ITPR2* gene (I2KO) in CD4^+^ and CD8^+^ T cells (Fig. S5a, b). Ablation of *ITPR2* resulted in significantly decreased transcription of *IFNG* and *IL2* in TCR-activated T cells (Fig. S5c). Flow cytometric analysis confirmed the reduction of IFN-γ in I2KO CD4^+^ and CD8^+^ T cells (Fig. S5d, e), highlighting the importance of InsP_3_R2-mediated Ca^2+^ signaling in human T cells. The unchanged IL-2 protein levels (Fig. S5f) suggest the involvement of additional regulatory mechanisms, such as alternative translational control of IL-2 [45]. In alignment with murine T cells (Fig. S4b), the addition of TAZ markedly upregulated the expression of *ITPR2* in activated human T cells (Fig. S5g). While TAZ reduced the expansion of TCR-activated CD4^+^ T cells, ablation of *ITPR2* partially but significantly rescued the expansion of TAZ-treated T cells (Fig. S5h). These data indicate that EZH2 regulates human T-cell survival by modulating *ITPR2* expression. Although short-term EZH2 inhibition did not affect cytokine production in TCR-activated WT T cells (Fig. 5E, F), TAZ increased IFN-γ production in I2KO CD4^+^ and CD8^+^ T cells (Fig. S5e) and reduced IL-2 production in I2KO CD8^+^ T cells (Fig. S5f). Thus, the EZH2-InsP_3_R2 axis plays a conserved role in regulating human T-cell expansion, whereas its impact on effector cytokine production varies with cellular context.

**Fig. 7:**
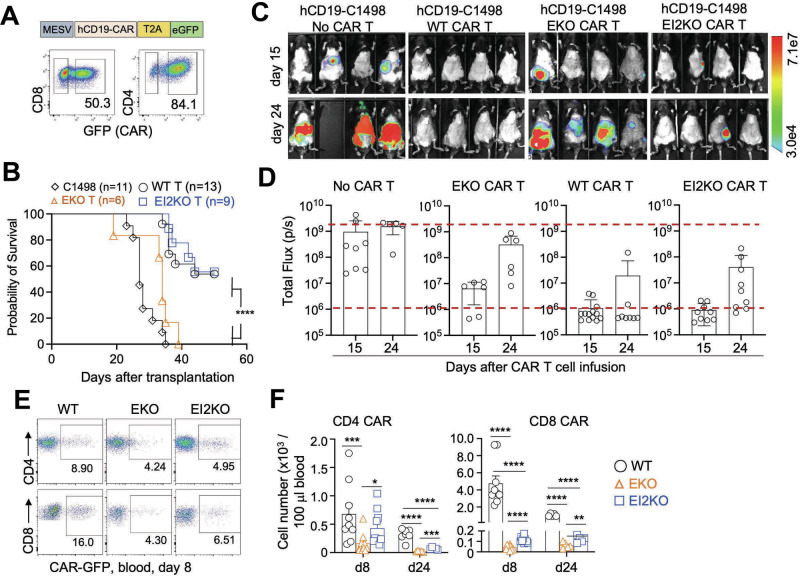
*Itpr2* ablation in EKO CAR-T cells restores their capacity to control leukemia. EI2KO and EKO CAR-T cells, along with WT controls, were intravenously infused into tumor-bearing mice one day post-tumor implantation. **A** Schematic representation of the mouse hCD19-CAR construct. **B** Survival probability across different treatment groups. **C** Representative images of tumor luciferase activity detected with live animals in vivo imaging system (IVIS) on days 15 and 24 post-implantation. **D** Summarized photon flux reflecting tumor growth dynamics in the tumor-only control group (*n* = 8), WT CAR-T cells (*n* = 13), EKO CAR-T cells (*n* = 6) and EI2KO CAR-T cells (*n* = 9) treatment groups. **E**, **F** Equal numbers (1 × 10^6^ cells) of WT, EKO and EI2KO CAR CD4^+^ and CD8^+^ T cells were transferred into hCD19-C1498 AML-bearing B6 mice. Peripheral blood was harvested 8 and 24 days after infusion of CAR-T cells for immunophenotyping and function assay. One-way ANOVA followed by post-hoc tests was performed for pairwise comparisons among multiple groups. *p < 0.05, **p < 0.01, ***p < 0.001, ****p < 0.0001

In the context of allo-HSCT, both host antigen-specific T cells and nonreactive donor T cells are activated to proliferate in lymphopenic hosts [46–48]. To define the impact of the EZH2-InsP_3_R2 axis in antigen-specific T cells, we employed CAR-T cells that were directed against a specific antigen. We cloned human CD19 (hCD19)-directed BBz CARs into an MSCV retroviral vector and produced murine hCD19-BBz CAR-T cells (Fig. 7A). B6 mouse-derived C1498 AML cells were transduced with lentivirus encoding hCD19 to generate hCD19-C1498 cells (Fig. S6a). EI2KO CAR-T cells exhibited similar cytotoxic effects against hCD19-C1498 cells (Fig. S6b–d). However, compared with WT cells, lower frequencies of EI2KO CD8^+^ CAR-T cells produced IFN-γ (Fig. S6e, f), which is potentially attributable to decreased Ca^2+^ signals.

To examine the ability of WT and EI2KO CAR-T cells to eliminate AML cells, we transplanted hCD19-C1498 cells into B6 mice, followed by the infusion of WT and EI2KO CAR-T cells one day later. EI2KO and WT CAR-T cells showed a similar capacity to control leukemia progression in AML-bearing B6 mice, with ~60% surviving 55 days after CAR-T-cell infusion (Fig. 7B). In vivo imaging revealed that all the mice treated with EKO CD19-BBz CAR-T cells died from AML (Fig. 7C, D). These data suggest broad implications of the EZH2-InsP_3_R2 axis in the regulation of antigen-specific T-cell responses.

### InsP_3_R2-mediated Ca^2+^ signaling induces acute phase defects in *Ezh2*-deficient CAR-T cells

**Fig. 8:**
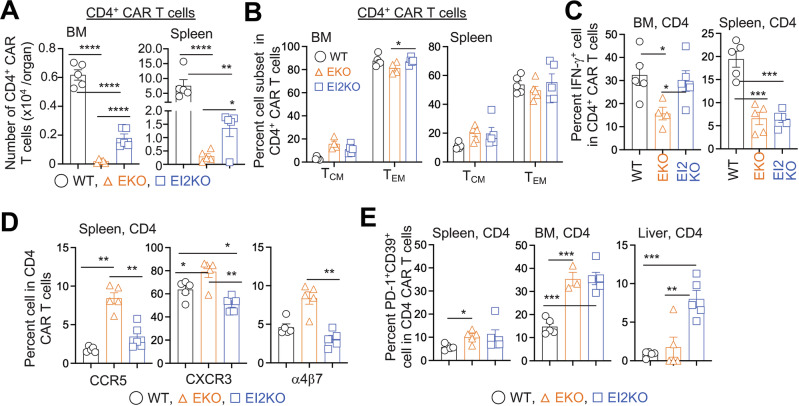
InsP3R2-mediated Ca^2+^ signals induce acute phase defects of *Ezh2*-deficient CAR-T cells. Equal numbers (1 × 10^6^ cells) of WT, EKO and EI2KO CAR CD4^+^ and CD8^+^ T cells were transferred into hCD19-C1498 AML-bearing B6 mice. Spleen, LN, BM and liver were harvested 10 days after infusion of CAR-T cells for CAR-T cells immunophenotyping and function assay. **A** Graphs show the number of CD4^+^ CAR-T cells in BM and spleen. **B** Graphs show the percentage of T_CM_ (CD44^+^CD62L^+^) and T_EM_ (CD44^+^CD62L^-^) in CD4^+^ CAR-T cells in the BM and spleen. **C** Graphs show the percentage of IFN-γ^+^ cells in CD4^+^CAR-T cells in the BM and spleen. **D** Graphs show the percentage of CCR5^+^, CXCR3^+^ and α4β7^+^ cells in CD4^+^ CAR-T cells in spleen. **E** Graphs show the percentage of PD-1^+^CD39^+^ cells in CD4^+^ CAR-T cells in spleen, BM and liver. One-way ANOVA followed by post-hoc tests was performed for pairwise comparisons among multiple groups. *p < 0.05, **p < 0.01, ***p < 0.001, ****p < 0.0001

To understand the mechanism by which *Itpr2* deletion rescued the antitumor activity of EKO CAR-T cells, we transferred equal numbers (1 × 10^6^ cells) of WT, EKO and EI2KO CD4^+^ and CD8^+^ hCD19-CAR-T cells into hCD19-C1498 AML-bearing B6 mice. WT CD4^+^ and CD8^+^ hCD19- CAR-T-cell numbers peaked in the peripheral blood (PB) as early as 8 days after transfer and declined by day 24 (Fig. 7E, F). Compared with WT CAR-T cells, EKO hCD19-CAR-T cells produced 4.3- and 15.0-fold fewer CD4^+^ CAR-T cells in the PB on day 8 and day 24, respectively (Fig. 7F), confirming the essential role of EZH2 in promoting the expansion of antigen-specific T cells [18–20, 22, 32]. Compared with EKO T cells, EI2KO CAR-T cells significantly increased the numbers of both CD4^+^ and CD8^+^ CAR-T cells in the PB at 8 and 24 days after infusion (Fig. 7F) and generated significantly more CAR-T cells in the BM and spleen at 10 days (Figs. 8A and S7a). These data suggest that the conditional deletion of *Itpr2* can rescue the survival of antigen-specific EKO T cells and restore their antitumor activity.

Intriguingly, compared with their WT counterparts, EI2KO CAR-T cells had significantly lower numbers of CD4^+^ and CD8^+^ CAR-T cells in the PB at 8 and 24 days after infusion (Fig. 7F) and in the BM and spleen at 10 days after infusion (Figs. 8A and S7a). This discrepancy in CAR-T-cell numbers between WT- and EI2KO-CAR-T cells might stem from an incomplete blockade of Ca^2+^ signaling in EI2KO CAR-T cells due to compensation from other InsP_3_R subunits.

We further assessed whether the conditional deletion of *Itpr2* in EKO CAR-T cells affects their effector differentiation and function. Compared with EKO CD4^+^ CAR-T cells, EI2KO CD4^+^ CAR-T cells produced a higher frequency of T_EM_ cells and IFN-γ-producing effector cells in the BM where leukemia cells were located (Figs. 8B, C and S7b). The loss of *Itpr2* in EKO T cells also led to a reduction of molecules critical for T-cell migration (CCR5, CXCR3 and α4β7) (Fig. 8D), which is consistent with decreased generation of Ca^2+^ signals [6, 31]. Thus, deletion of *Itpr2* in EKO hCD19-CAR-T cells significantly improved their expansion early during the response to leukemia, with greater protection on CD4^+^ CAR-T cells.

PD-1^+^CD39^+^ T cells are terminally exhausted cells (term-T_EX_) [49–51]. Compared with WT CD4^+^ hCD19-CAR-T cells, EI2KO CAR-T cells produced a 1.8-fold and 3.5-fold greater frequency of PD-1^+^CD39^+^ T cells in the BM and liver, respectively (Fig. 8E). This terminal exhaustion likely results from the loss of EZH2. However, this cannot be detected in the presence of STIM1 or InsP_3_R2 in *Ezh2*-null cells due to precocious death. Given that Ca^2+^ signaling-activated NFAT promotes exhaustion [52], our findings from these EI2KO T cells reveal previously unknown roles for EZH2 in suppressing T-cell exhaustion upon chronic antigen exposure via the suppression of *Itpr2* expression.

Interestingly, compared with EKO CD8^+^ hCD19- CAR-T cells, EI2KO CD8^+^ hCD19- CAR-T cells had 3.4 to 4.4-fold greater numbers of CD44^+^CD62L^-^ T_EM_-phenotype cells in the BM and spleen 10 days after transplantation (Fig. S7c). EI2KO CD8^+^ hCD19-CAR-T cells retained the ability to produce IFN-γ^+^ effector cells but were significantly lower than their WT counterparts (Fig. S7d–f), which is potentially attributable to a critical role for InsP_3_R2 in effector differentiation that cannot be compensated for in EI2KO CD8^+^ T cells. EI2KO CD8^+^ hCD19- CAR-T cells produced significantly higher frequencies of PD-1^+^CD39^+^ term-T_EX_-like cells in the spleen and liver than their WT counterparts (Fig. S7g). This role of EZH2 in limiting CAR-T-cell exhaustion could explain the inability of *Itpr2* loss to restore EKO CAR-T-cell persistence later during the tumor control phase.

### Inhibition of Ca^2+^ signaling enhances EZH2 function in CAR-T cells

Emerging evidence indicates that the function of EZH2 is dynamically regulated in normal T cells during immune responses [53–55]. Differentiated T cells, particularly short-lived T_E/EM_ cells, are associated with a decline in immune function, increased susceptibility to infections and impaired antitumor immunity [56, 57]. EZH2 function is downregulated in T_EM_ cells, as evidenced by the loss of the H3K27me3 marker at the promoter regions of *Prdm1* and *Eomes*, leading to the upregulation of these transcription factors and subsequent terminal differentiation [18, 40]. We found that AKT activation profoundly reduced EZH2 function in activated murine CD8^+^ T cells [18]. A recent study revealed that tonic CAR signaling, in which CAR-T cells are persistently activated, leads to EZH2 dysfunction and impaired antitumor activity [58].

Given that EZH2 and Ca^2+^ signaling interdependently coordinated T-cell differentiation (Figs. 1, 4), we hypothesized that modulating Ca^2+^ signaling in CAR-T cells could enhance EZH2 functionality. We used two strategies to test this hypothesis. We first added the CNI tacrolimus (Tac) to cultured CAR-T cells to assess whether inhibition of the calcineurin/NFAT pathway, which is associated with T-cell exhaustion [8, 59], may affect their EZH2 functionality. Compared with the PBS control, Tac-treated CAR-T cells significantly increased the levels of H3K27me3 (Fig. S8a). This was accompanied by decreased expression of *Ifng*, *Tbx21* and *Eomes*, whose expression is repressed by EZH2 [18, 21, 22, 40] and upregulation of *Id3* expression (Fig. S8b), which is activated by EZH2 [18]. This was verified through pharmacological blockade of STIM1-mediated SOCE using BTP2 (YM-58483) [60]. We found that BTP2 significantly increased the level of H3K27me3 in CAR-T cells, which was accompanied by decreased expression of *Ifng* and *Tbx21* (Fig. S8c, d). Thus, inhibiting Ca^2+^ signaling in cultured CAR-T cells can significantly increase EZH2 activity.

We next used a cell stress model in which a limited number of CAR-T cells failed to eliminate leukemia, as previously described [61], to evaluate whether enhancing EZH2 functionality through a reduction in Ca^2+^ signaling improved CAR-T-cell antitumor activity (Fig. S9a). Adoptive transfer of Tac-primed CAR-T cells significantly enhanced leukemia control compared with that of PBS-primed CAR-T cells, as shown by enhanced inhibition of leukemia growth and increased overall survival rates (Fig. S9b, c). Since in vivo CNI treatment restricts the terminal exhaustion of alloreactive T cells, leading to chronic GVHD [8, 9], we hypothesized that pretreatment of CAR-T cells with Tac could reduce their differentiation into term-T_EX_-like cells, thereby enhancing their antitumor activity. We isolated CAR-T cells from the spleen, liver and BM of hCD19-C1498 leukemia-bearing mice 9 days after the infusion of Tac-CAR-T cells and PBS-CAR-T cells, corresponding to the peak expansion phase of WT CAR-T cells in vivo (Fig. 7F), which correlated with the potent suppression of leukemia growth (Fig. 7C, D). We observed that ex vivo Tac-primed CAR-T cells significantly increased the frequency of PD-1^+^CD39^-^ progenitor effector cells but decreased the frequency of PD-1^+^CD39^+^ term-T_EX_-like cells in the spleen and liver compared with those in the PBS control group (Fig. S9d, e). These results explain the enhanced potency of Tac-treated CAR-T cells to eliminate AML cells in vivo.

## Discussion

During the early/intermediate phases of the immune response, Ca^2+^ signals are crucial for T-cell activation, proliferation and metabolism; during the later effector differentiation phase, excessive or prolonged Ca^2+^ signals lead to T-cell death and dysfunction [7, 24, 28, 62]. The epigenetic mechanisms underlying shifts in Ca^2+^ signal homeostasis after T-cell activation are not fully understood. Our findings underscore the essential role of coordinated EZH2 activity and Ca^2+^ signaling in governing these processes. This dynamic interplay between EZH2 and Ca^2+^ signaling is vital for maintaining a balance that prevents T cells from falling into either “death” or “exhaustion”. Mechanistically, EZH2 repressed intracellular Ca^2+^ responses, in part by repressing the expression of the ER Ca^2+^ release channel InsP_3_R2, thereby promoting the survival of activated T cells during late effector differentiation. In parallel, EZH2 silenced the expression of Ca^2+^ signaling-activated genes responsible for terminal differentiation and exhaustion. Conversely, prolonged Ca^2+^ signaling reduced T-cell EZH2 functionality. Transient blockade of the calcineurin/NFAT pathway during ex vivo expansion of antigen-specific T cells could preserve EZH2 functionality, thereby augment antitumor responses in vivo. Together, EZH2 and Ca^2+^ signaling serve mutually opposing roles in fine-tuning the gene programs required for productive T-cell responses.

The interdependence between EZH2-regulated gene programs and Ca^2+^ signals may have significant implications for understanding GVHD pathology following CNI treatment. Alloreactive T cells with memory potential are crucial for sustaining GVHD [49, 63–65]. We have shown that EZH2 and its target protein ID3 are required for sustaining alloreactive T cells in GVHD tissues [20, 66]. Treatment with CNIs has been the standard GVHD prophylactic regimen in allo-HSCT clinics [3]. CNIs suppress the activation and effector differentiation of alloantigen-activated T cells through repressing transcription of the Ca^2+^/CN-dependent gene program in T cells. Intriguingly, while CNI treatment suppresses T-cell activation, it promotes the expansion of host-reactive memory-like T cells that mediate chronic GVHD [9]. Our studies suggest that the coordinated action of EZH2 and Ca^2+^ signaling pathways is vital for maintaining alloreactive T cells critical for persistent injury to host tissues. We propose that novel strategies capable of selectively heightening intracellular Ca^2+^ signals in alloreactive T cells may be developed to induce durable control of GVHD, potentially offering an alternative to conventional CNI treatment.

One key finding of our study is the identification of a causal regulatory role of EZH2 in controlling Ca^2+^ signaling pathways in T cells. The functional links between EZH2 and Ca^2+^ signaling have not previously been defined. We found that EZH2 restricted STIM1 sensitivity such that its deficiency led to STIM1 polarization in the absence of cognate antigen and enhanced SOCE upon TCR stimulation. Notably, this phenotype was not associated with increased *Stim1* transcription, suggesting that EZH2 modulates upstream pathways that control STIM1 sensitivity. Mechanistically, our data support that EZH2 restrains Ca^2+^ signaling in part by repressing the ER Ca^2+^ release channel InsP_3_R. On one hand, subsets (T_E/EM_ and T_CM_) with increased EZH2-associated repression during differentiation exhibited higher H3K27me3 enrichment at the promoter region of *Itpr2* than T_N_ cells. Accordingly, genetic or pharmacological EZH2 inhibition in TCR-activated T cells increased *Itpr2* transcription, which was accompanied by reduced H3K27me3 deposition at *Itpr2* promoter regions. These data indicate that EZH2 directly represses *Itpr2* expression to prevent Ca^2+^ signaling hyperactivation.

Although we did not directly profile EZH2 occupancy at the *Itpr2* locus, H3K27me3 is a well-established functional readout of PRC2-mediated repression [67]. The concordant reduction in H3K27me3 and increase in *Itpr2* expression upon *Ezh2* deletion strongly support a model in which EZH2 maintains repressive chromatin at this locus. We acknowledge that additional chromatin-modifying mechanisms may also contribute, and future studies are warranted to define the dynamics of EZH2 recruitment and H3K27me3 turnover at *Itpr2* regulatory loci. At the transcriptional level, we observed a significant overlap of DEGs between ESKO Th1 cells and alloreactive SKO T cells from GVHD mice. While the loss of *Stim1* alone suppressed effector-associated gene expression, the simultaneous deletion of *Ezh2* in SKO Th1 cells restored these effector pathways. These findings indicate that EZH2 counterbalances Ca^2+^ signaling-driven transcriptional programs in effector T cells, supporting a causal role for Ca^2+^ signaling in shaping EZH2-dependent T-cell function. Given the broad and context-dependent roles of both EZH2 and Ca^2+^ signaling in T cells, it remains possible that Ca^2+^ signaling may act as an accompanying or modulatory mechanism of altered EZH2 activity in other immune settings not addressed here.

Increased intracellular Ca^2+^ accumulation may cause cell death [24, 28, 29]. It has been shown that SOCE triggers T-cell death by inducing FasL and TRAIL expression and activates cell intrinsic apoptosis by upregulating the expression of proapoptotic genes (such as Noxa and Bak), which facilitate cytochrome-C release from mitochondria [7, 24, 28, 29]. Our studies and others have demonstrated that loss of *Ezh2* in murine T cells causes increased expression of *Bcl2l11*, *Fas*, *Tnfr1a*, and *Trail* (*Tnfsf10*). However, blockade of these molecules does not completely prevent cell death [20, 23]. Thus, heightened Ca^2+^ signaling may mediate cell death in activated *Ezh2*-null T cells via mechanisms independent of these cell death receptors and intrinsic proapoptotic molecules. For example, inhibition of EZH2 in human T cells is associated with DNA damage-induced cell death [68]. While death induction can effectively suppress T-cell-mediated pathogenic responses such as GVHD, this occurs at the cost of irreversible impairment of protective T-cell immunity against tumors and pathogens [69, 70]. In addition to death induction, Ca^2+^ signaling activates NFAT, TOX and NR4A families, which drive exhaustion under persistent antigen stimulation [71, 72]. Enhanced exhaustion of alloreactive T cells has also been implicated in the attenuation of GVHD [8, 49, 66].

Our findings reveal that the interaction between EZH2 activity and Ca^2+^ signaling balances immune response outcomes, specifically by regulating the balance between cell death and exhaustion. We observed that deleting *Itpr2* in EKO T cells (generating EI2KO cells) unmasked a T-cell exhaustion phenotype (PD-1^+^CD39^+^), provided that Ca^2+^ signaling-induced cell death was partially reduced. T-cell exhaustion is defined as a decreased capacity for effector function and proliferation following chronic antigen exposure. However, exhausted T cells can potentially be reinvigorated after the blockade of PD-1 and/or CTLA4 expression [71, 73, 74]. Conversely, T-cell death effectively suppresses inflammatory T-cell responses such as those associated with GVHD, but this occurs at the cost of irreversibly impairing T-cell immunity against tumor cells and pathogens. These data suggest that EZH2 may not only suppress excessive Ca^2+^ signaling-induced T-cell death but also limit T-cell exhaustion through mechanisms beyond the inhibition of excessive ITPR2-Ca^2+^ signaling.

Our studies also demonstrated that Ca^2+^ signaling can modulate EZH2 functionality in antigen-reactive T cells. T-cell dysfunction, including memory potential loss and exhaustion, is a major limitation of CAR-T-cell therapy efficacy [52, 58, 75–82]. CAR-T-cell dysfunction, particularly exhaustion, has been linked to calcium signaling, most notably through hyperactivation of the Ca^2+^/CN-dependent NFAT pathway [52, 72, 77, 83, 84]. However, the underlying epigenetic mechanisms remain poorly defined. During immune responses, antigen-activated T cells rapidly upregulate EZH2 expression following stimulation, yet EZH2 function can be profoundly decreased during effector differentiation. We have demonstrated that antigen stimulation-induced AKT activation leads to phosphorylation of the EZH2 protein at serine 21 and subsequent decreases in the protein stability and function of EZH2 [18]. Furthermore, many other factors (e.g., p38 MAP kinase and GSK3-β) may phosphorylate the EZH2 protein to reduce EZH2 activity [85]. Recent studies have suggested that calcium/calmodulin-dependent protein kinase II alpha (CAMK2A), a key Ca^2+^ signaling molecule, can reduce EZH2 functionality [86]. Given the crucial role of Ca^2+^ signaling in activating the PI3K/AKT, p38 MAPK and CAMK2 signaling pathways, these observations collectively support a model in which sustained Ca^2+^ signaling could downregulate EZH2 activity in activated T cells. Indeed, we found that inhibition of Ca^2+^ signaling during ex vivo CAR-T-cell generation enhanced EZH2 function and was associated with improved in vivo tumor control efficacy. These findings thus support that reduction of EZH2 function contributes to Ca^2+^/CN-dependent NFAT-driven CAR-T-cell dysfunction, thereby signify the translational value of priming CAR-T cells with Ca^2+^ signaling inhibition. Future studies investigating the molecular pathways through which Ca^2+^ signaling modulates EZH2 function will be critical for refining strategies that improve CAR-T-cell efficacy while maintaining an acceptable safety profile.

Our manufacturing-based strategy mitigated systemic immunosuppression by restricting Tac exposure to the ex vivo expansion phase. However, since the resulting enhancement in CAR-T-cell expansion and persistence may correlate with an increased risk of cytokine release syndrome and on-target off-tumor toxicity, future clinical translation requires a robust risk management framework focused on these specific safety parameters.

In summary, since CNI treatment reduces alloreactive T-cell responses during the GVH reaction and since antigen-driven T cells may reduce EZH2 functionality during effector proliferation and differentiation [18], future studies should determine the mechanisms by which excessive and prolonged Ca^2+^ signaling downregulates EZH2 expression and functionality in antigen-specific T cells. Additionally, InsP_3_Rs play a pivotal role in regulating intracellular Ca^2+^ homeostasis and dynamics by controlling Ca^2+^ flux from the ER into the cytosol and mitochondria [87]. Defining the molecular events through which EZH2 modulates the expression/function of InsP_3_Rs in antigen-driven T cells is important. The results of these studies may provide novel insights into the epigenetic mechanisms that control Ca^2+^ responses critical for productive T-cell immunity in the context of alloimmunity, autoimmunity, chronic infection and tumor immunotherapy.

## Materials and methods

### Mice

C57BL/6 (B6, H-2b, CD45.1^-^CD45.2^+^), B6/SJL (H-2b, CD45.1^+^CD45.2^-^), and BALB/c mice were purchased from The Jackson Laboratory. Balb/b mice were maintained in-house. F1 B6 mice (H-2b, CD45.1^+^ CD45.2^+^) were generated by crossing C57BL/6 mice with B6/SJL mice. B6 background *Ezh2*^fl/fl^, *Itpr1*^fl/fl^, *Itpr2*^fl/fl^, *Itpr3*^fl/fl^ and *Stim1*^fl/fl^ mice were bred in house. These mice were crossed with CD4-Cre or CD2-Cre mice to produce CD4-Cre^+^*Ezh2*^fl/fl^ B6 mice, CD2-Cre^+^*Ezh2*^fl/fl^ .*Stim1*^fl/fl^ B6 mice, *Ezh2*^fl/fl^. *Stim1*^fl/fl^ B6 mice, CD4-Cre^+^*Ezh2*^fl/fl^*Itpr2*^fl/fl^ B6 mice, CD4-Cre^+^*Itpr1*^fl/fl^ B6 mice, CD4-Cre^+^*Itpr2*^fl/fl^ B6 mice, and CD4-Cre^+^*Itpr3*^fl/fl^ B6 mice. Experimental protocols were approved by the Institutional Animal Care and Use Committees of Temple University and Hackensack Meridian Health.

### Murine cell preparation and culture

CD4^+^ and CD8^+^ T cells were isolated from the spleens and LNs of age-matched WT and KO mice using CD4 and CD8 microbeads (Miltenyi Biotech). Naïve T cells were further purified by depleting CD44^+^ T cells using biotin-conjugated anti-CD44 (BioLegend) and streptavidin Dynabeads (Miltenyi Biotech). TCD-BMs were prepared as described previously [63]. For Cre^- ^T cells with floxed genes, genes were deleted through TAT-Cre treatment under homeostatic conditions (IMDM containing 10% FBS supplemented with 10 ng/ml IL2, 10 ng/ml IL7, and 10 ng/ml IL15). CD4^+^ T cells were stimulated with anti-CD3/CD28-conjugated Dynabeads (Gibco) and cultured in Th1 polarization medium of IMDM containing 10% FBS supplemented with 10 ng/ml IL2, 10 ng/ml IL12 and 10 μg/ml anti-IL4. CD8^+^ T cells were stimulated with anti-CD3/CD28-conjugated Dynabeads (Gibco) and cultured in IMDM containing 10% FBS supplemented with 10 ng/ml IL2. In some experiments, various doses of tazemetostat (MedChemExpress) were added to cultured murine T cells to reduce H3K27me3 levels.

### Murine GVHD models

Mice underwent bone marrow transplantation as described previously [64]. To induce GVHD, we gave BALB/c mice 850 cGray irradiation from an X-ray source followed by transplantation with donor B6 TCD-BM cells (5 × 10^6^), with or without the addition of CD4^+^ and CD8^+^ T cells. Recipients were monitored for survival and clinical signs of GVHD using a scoring system that includes weight loss, posture, mobility, fur and skin impairment. The epithelial organs (skin, liver, and intestine) were harvested for histological examination (Pathology Core, Fox Chase Cancer Center).

### Antibodies (Abs), flow cytometry analysis and cell sorting

Single-cell lymphocyte suspensions from the spleen, mLNs and liver were separated as described previously [88]. All antibodies used for immunofluorescence staining were purchased from eBioscience (San Diego, CA), BioLegend (San Diego, CA) or BD Biosciences (San Jose, CA). Cells were stained with appropriate concentrations of Abs. Dead cells were excluded using Fixable Viability Dye from eBioscience (San Diego, CA). Flow cytometry analyses were performed using BD LSRII, LSRFortessa or FACSymphony A3 cytometers (BD Bioscience, CA). Cell sorting was performed with BD Aria (BD Bioscience, CA).

### Mouse acute myeloid leukemia model

hCD19-C1498 acute myeloid leukemia (AML) cells (0.5 × 10^6^ cells/mouse) were inoculated into 7–10-week-old B6 mice through the tail vein (i.v.) on the day of sublethal irradiation (day 0). Afterwards, the recipients received infusions of 1 × 10^6^ CAR-T cells on day 1. The survival of the mice was monitored until the experimental or humane endpoints were reached. CAR-T-cell phenotype and function were evaluated ex vivo using flow cytometry on day 10 postinfusion. Leukemia progression was assessed using in vivo bioluminescence with an IVIS Lumina X5 Imaging System (Perkin Elmer, MA). For imaging, the mice were administered 1.5 mg of D-luciferin (Pierce) intraperitoneally prior to the imaging sessions.

### Retrovirus and mouse CAR-T-cell production and transplantation

hCD19-41BB-CAR was synthesized using codon optimization and cloned into the MSCV-EF1α-eGFP vector. Platinum-E cells were transfected with the retroviral CAR plasmid and Ecopac packaging plasmid using TransIT®-LT1 Transfection Reagent (Mirus, WI) to produce the retrovirus. T cells were activated and transduced with hCD19-41BB-eGFP CAR retrovirus at a multiplicity of infection (MOI) of 3–5, 24 and 48 h post-activation. hCD19-41BB-eGFP CAR-transduced T cells were cultured for an additional three days, harvested and injected (i.v.) into hCD19-C1498 leukemia-bearing B6 mice. In some experiments, hCD19-41BB-eGFP CAR-T cells (CD4^+^ and CD8^+^) of B6 mouse origin were treated with tacrolimus (Tac, 50 ng/ml) or PBS from day 3 to day 6 after TCR activation. The resultant Tac-CAR-T cells and PBS-CAR-T cells were harvested on day 6, thoroughly washed to remove Tac, and subsequently transferred (i.v.) into hCD19-C1498 leukemia-bearing B6 mice. Survival and leukemia growth were monitored over time.

### CAR-T-cell in vitro cytotoxicity assay

Human CD19-expressing C1498 cells (hCD19-C1498) were seeded at a density of 1 × 10^5^ cells per well in a 24-well plate. These cells were cocultured with CAR-T cells at effector-to-target (E: T) ratios of 1:1, 1:3, 1:9, and 1:27. Additionally, wells containing only tumor cells served as controls. The production of interferon-gamma (IFN-γ) was assessed by flow cytometry 12 to 24 h after the initiation of coculture. At specific time points (day 1, day 2, and/or day 3 post coculture), an equivalent volume of cell suspension was collected from each well for live tumor cell count comparisons, and tumor-only wells were used as controls. Cytotoxicity was calculated using the following formula: Cytotoxicity (%) = (1-Coculture group tumor cell count/Tumor-only group tumor cell count) X 100.

### Calcium flux assay

Calcium influx assay was performed as previously described [30, 89]. In some experiments, T cells were activated with anti-CD3/CD28 antibody for 20 h, then plated on coverslips and placed in cation-safe solution (107 mM NaCl, 7.2 mM KCl, 1.2 mM MgCl_2_, 11.5 mM glucose, 20 mM HEPES-NaOH, 1 mM CaCl_2_, pH 7.2) and loaded with fura-2/acetoxymethylester (2 μM) for 30 min at 24 °C. The cells were subsequently washed, and the dye was allowed to de-esterify for a minimum of 30 min at 24 °C. Ca^2+^ measurements were performed using a Leica DMI 6000B fluorescence microscope controlled by Slidebook Software (Intelligent Imaging Innovations; Denver, CO). In some experiments, CR-activated T cells (1 X 10^6^) were incubated with 5 µM Calbryte™ 590 AM (AAT Bioquest) in Ca^2+^ and Mg^2+^-free HHBS buffer (Hank’s buffer with 20 mM HEPES) containing 0.04% Pluronic F-127 (Thermo Fisher) for 30 min at 37 °C. After extensive washing in HHBS without Ca^2+^ and Mg^2+^, the cells were incubated with biotinylated anti-CD3 antibodies (5 µg/ml for CD4^+^ T cells and 1 µg/ml for CD8^+^ T cells; 15–30 min, room temperature) and washed twice. The cells were then resuspended in HHBS supplemented with Ca^2+^ and Mg^2+^ and immediately analyzed using a Fortessa flow cytometer. Calbryte 590 AM activation and detection involved excitation at 581 nm and emission at 593 nm as calcium indicators. Each sample was recorded for 30–60 seconds (baseline) before 1 µg/ml streptavidin was added to crosslink the anti-CD3ε antibody, followed by another 5 min of recording. In some experiments, T cells were treated with 0.5 µM thapsigargin to measure Ca^2+^ flux for 20 min before 1 µg/ml streptavidin was added, and the influx of extracellular Ca^2+^ was monitored over the next 5 min. The difference between the maximal and minimal signal intensity (peak value) is measured for ratiometric detection.

### Immunofluorescence staining

Naïve WT and EKO CD8^+^ T cells were freshly isolated from mice, cytospun onto slides, and fixed with 4% paraformaldehyde for 15 min at room temperature. Fixed cells were washed with PBST (0.01% Triton X-100) and stained with anti-STIM1 antibody (1:100; Cell Signaling #5668) overnight at 4 °C. After being washed with PBST 3 times, the cells were incubated with fluorophore-conjugated anti-rabbit IgG (1:2000, Invitrogen, # A-11008) for 1 h at room temperature, washed with PBST and mounted with antifade mounting medium (Vector Laboratories, # H-1000-10).

### Real-time quantitative PCR

Total RNA was extracted from cultured or sorted T cells with TRIzol (Invitrogen Life Technologies), digested with DNase to remove genomic DNA and reverse transcribed into cDNA using a Superscript IV VILO cDNA synthesis kit (Invitrogen Life Technologies). Real-time quantitative PCR was performed with SYBR Green PCR master mix with ROX as the reference dye (Roche) on a QuantStudio 5 Real-time PCR instrument. The gene expression level was calculated relative to that of the endogenous control 18S rRNA.

### Bulk RNA sequencing and analysis

Total RNA was extracted with a Qiagen RNeasy Mini Kit. Directional mRNA libraries were prepared using the poly(A) enrichment pipeline with the NEBNext Ultra II Directional RNA Library Prep Kit for Illumina (New England Biolabs, Inc.) following the manufacturer’s protocol. Libraries were sequenced on Novaseq in paired-end mode with a read length of 150 nucleotides by Novogene. For the GVHD data, sequence reads were aligned to the mouse mm10 genome using TopHat [90]. Cuffdiff was used to statistically assess expression changes in quantified genes across different conditions [91]. Genes with a false discovery rate (FDR) of ≤5% and a fold change of ≥2 were considered to be differentially expressed for the EKO vs WT comparison, whereas an FDR cutoff of ≤20% and a fold change of ≥1.5 were used for the SKO vs WT comparison.

For the dataset derived from ESKO T cells, the reads were aligned to the mm10 genome with STAR. The number of raw counts for each known gene from the RefSeq database was determined using HTSeq-count from the HTSeq package. Differential expression across different conditions was assessed for statistical significance using the R/Bioconductor package DESeq2. Genes with an FDR ≤ 0.05 and a fold change ≥ 2 were considered significant. The enriched canonical pathways and gene interaction networks of significant genes were analyzed with the use of QIAGEN IPA (QIAGEN Inc., https://digitalinsights.qiagen.com/IPA).

### CUT&RUN and qPCR analysis

CUT&RUN qPCR was used to examine the enrichment of H3K27me3 in the regulatory regions (promoters and enhancers) of *Itpr2*. The CUT&RUN reaction was performed following the EpiCypher protocol with minor modifications [92]. Briefly, 5 × 10^5^ per reaction cultured CD8^+^ and CD4^+^ T cells were harvested and immobilized by binding to activated Concanavalin A (ConA)-coupled magnetic beads (BANGS LABORATORIES INC). Immobilized cells were permeabilized in 0.01% digitonin-containing antibody buffer (20 mM HEPES, pH 7.5; 150 mM NaCl; 0.5 mM spermidine, 1X Roche cOmplete^TM^ EDTA-free protease inhibitor; 2 mM EDTA), added SNAP-CUTANA^TM^ K-MetStat Panel spike-in (EpiCypher), then incubated with rabbit IgG (0.5 μg) or H3K27me3 antibody (Cell Signaling #9733, 1:50) overnight at 4 °C on a nutator. The unbound antibody was removed by washing with digitonin buffer (20 mM HEPES, pH 7.5; 150 mM NaCl; 0.5 mM spermidine; 1X Roche cOmplete^TM^ EDTA-free protease inhibitor; 0.01% digitonin). Cells were incubated with protein A/G-micrococcal nuclease (pAG-MNase) (kindly produced by Prof. Hai-Hui Xue’s laboratory, with a prokaryotic expression plasmid from Addgene, 123461) for 30 min at room temperature. After the unbound pAG-MNase was removed with digitonin buffer, the antibody-bound MNase was activated by the addition of CaCl_2_ (at a final concentration of 2 mM). Chromatin was digested for 4 h at 4 °C on a nutator and then quenched with stop buffer (340 mM NaCl; 20 mM EDTA, 4 mM EGTA, 50 µg/ml RNase A; 50 µg/ml glycogen). Cleaved chromatin was released by incubation at 37 °C for 10 min and then purified with an EpiCypher CUTANA^TM^ DNA Purification Kit. qPCR was performed using SYBR Green mix on purified DNA with K-MetStat spike-in as the internal control, which was then normalized to the IgG control.

### Western blot

Cells were harvested, pelleted and lysed in RIPA buffer. The total protein concentration was measured with a BCA protein assay (Pierce). Equal amounts of protein from each sample were denatured in Laemmli buffer containing 2-mercaptoethanol. The proteins were separated by 15% sodium dodecyl sulfate‒polyacrylamide gel electrophoresis and transferred to a nitrocellulose membrane. Blots were blocked in skim milk followed by overnight incubation with primary antibodies (Cell Signaling: H3K27me3, #9733; EED, #85322; SUZ12, #3737; EZH2, #5246; EZH1, #87528; Histone H3, #9715) at 4 °C. The next day, the blots were washed and incubated with an HRP-conjugated secondary antibody (Cell Signaling #7074) at room temperature for 1 h. The blots were then washed and developed using ECL plus (Perkin Elmer) and imaged with an AZURE Biosystems 600.

### CRISPR/Cas9 knockout (KO) of ITPR2 (ITPR2-KO) in human T cells

Two single guide RNA (sgRNA) sequences targeting ITPR2 were synthesized by Integrated DNA Technologies (IDT) (Table S2)). CD4^+^ and CD8^+^ T cells were isolated with MojoSort™. Primary human T cells were first isolated from thawed PBMCs using a negative selection method (MojoSort™ Human CD4/8 T-Cell Isolation Kit) and cultured with 10 ng/mL rhIL-2 and 5 ng/mL of rhIL-7 and rhIL-15 (Shenandoah). T cells were activated with Dynabeads Human T-Activator CD3/CD28 (Gibco, NY) at a 1:1 bead-to-cell ratio. On day 2, the Dynabeads were removed, and the cells were resuspended at 1 × 10^8^ cells/mL in Ingenio solution (Mirus, MA). The ribonucleoprotein (RNP) complexes were generated by incubating each sgRNA (5 µg per 10 × 10^6^ cells) individually with the Alt-R CRISPR-Cas9 nuclease V3 (IDT, 10 µg per 10 × 10^6^ cells) for 15 min at room temperature. RNP plus 16.8 pmol of electroporation enhancer (IDT) was electroporated into the cells using a BTX830 (Harvard Apparatus BTX) at 360 V and 1 ms; this process was followed by a second electrotransfer of 5 μg of gRNA 12 to 24 h later. The knockout efficiency was assessed via T7E1 mismatch assays and Sanger sequencing. The resultant WT and ITPR2-KO T cells were expanded with or without Taz treatment to assess proliferation and survival. Cells were collected 6 days post-Taz treatment for RNA extraction and gene expression analysis.

### Statistical analysis

Differences in survival between groups were determined with the log-rank test. Clinical scores were compared between groups using Student’s *t* test and the Mann‒Whitney test. Two-group mean comparisons were performed with Student’s *t* test; multiple-group mean comparisons were performed with one-way ANOVA. A *p* value less than 0.05 was considered to indicate a significant difference.

## Supplementary information

Combined supplemental figures and legends

Table S1. qPCR primers

Table S2. CRISPR primers

unprocessed images

## Data Availability

For information and requests for resources and reagents, please contact Yi Zhang (yi.zhang@hmh-cdi.org). The GEO IDs for each of the experiments are as follows: Super Series Accession: GSE275045 and GSE275046 (RNA-seq data).
