# Supplementary material for: EZH2 and intracellular Ca2+ signals interdependently coordinate alloreactive and CAR-T-cell responses
Source: Cell Mol Immunol. 2026 Apr 22;23(7):840–54. doi: 10.1038/s41423-026-01413-y (PMC13314966; doi:10.1038/s41423-026-01413-y)
Supplement: Supplementary file 1 — Combined supplemental figures and legends [file 41423_2026_1413_MOESM1_ESM.pdf]

SUPPLEMENTARY FIGURE LEGENDS

Supplementary Fig.1. The loss of Ezh2 in TCR-activated CD8$^+$ T cells leads to increased expression of genes associated with the activation of Ca^{2+} signals. a. In-silico analysis was performed on our published RNA-seq dataset (accession no. GSE76755). The chart lists the most significant activated and inhibited upstream regulators associated with genes altered in TCR-activated EKO CD8$^+$ T cells. **b**. Western blots show the protein levels of EZH1 and core components of PRC2 complex (SUZ12, EED, EZH2) in TCR-activated WT and EKO CD8$^+$ T cells. **c**. Bar graph shows the mRNA levels of *Stim1* and *Orai1* in TCR activated WT and EKO CD8$^+$ T cells. **d**. Naïve WT and EKO CD8$^+$ T cells were stained with anti-STIM1 followed by Alexa Fluor 647-anti-rabbit IgG.

Supplementary Fig.2. GVHD clinical scores of Balb/c recipients. **a**. As described in Fig.1a, ESKO CD8 T cells were activated with anti-CD3/CD28 antibody for 20 hours, loaded with fura-2 for 1 h and depleted ER Ca^{2+} stores with Thapsigargin (Tg, 2 μM) and monitored under a fluorescent microscope for Ca^{2+} oscillation. Each trace represents Ca^{2+} oscillation pattern in one cell. Data shown are representative of 3 independent experiments. **b.** As described in Fig.1b, lethally irradiated Balb/c mice were transplanted with naïve WT, EKO T cells, or SKO T cells (5X10^5 CD4$^+$ and 2X10^5 CD8$^+$ T cells). Clinical signs of GVHD were monitored over time. $^{**}p<0.01$, $^{***}p<0.001$.

Supplementary Fig.3. Induction of GVHD by CD2.Cre-ESKO T cells. a. Balb/c mice were given total body irradiation (TBI, 4.5 Gy on day -1 and 4 Gy on day 0) followed by infusion of 5X10^6 B6/SJL mouse (CD45.1$^+$CD45.2$^-$) TCD-BM alone or together with B6 (CD45.1$^-$CD45.2$^+$) naïve EKO T cells or CD2.Cre-ESKO T cells (5X10^5 CD4$^+$ and 2X10^5 CD8$^+$ T cells). Survival was monitored over time. **b.** As described in Fig.3g-h, WT, EKO, CD2.Cre-ESKO CD4$^+$ and CD8$^+$ T cells were activated using anti-CD3/CD28 antibodies in cultures. Flow plots show the dead cell frequency stained with Annexin 5 and 7-AAD 6 days after activation. **c-d.** Flow plots and bar graphs show the thymus development of CD2.Cre-ESKO mice and their littermate controls. Representative data of 3 independent experiments were shown. Statistical analysis was performed with the Log-rank (Mantel–Cox) test. $^{***}p<0.001$.

Supplementary Fig.4. I2KO T cells retain potent capacity to induce GVHD. a. CD8$^+$ T cells were activated and treated with different concentrations of TAZ. Cells were harvested 3 days after activation to examine the protein levels of H3K27me3 and EZH2 using western blot. **b.** qPCR was performed to examine the transcription of Itpr2 in activated CD8$^+$ and CD4$^+$ T cells treated with TAZ. **c.** Balb/c mice were given total body irradiation (TBI, 4.5 Gy on day -1 and 4 Gy on day 0) followed by infusion of 5X10^6 B6/SJL mouse (CD45.1$^+$CD45.2$^-$) TCD-BM alone or together with B6 (CD45.1$^-$CD45.2$^+$) naïve WT and I2KO T cells (5X10^5 CD4$^+$ and 2X10^5 CD8$^+$ T cells). Graph shows survival of Balb/c recipients. Statistical analysis was performed with the Log-rank (Mantel–Cox) test.

Supplementary Fig.5. ITPR2 ablation in human T cells partially rescued the deficiency caused by pharmacological inhibition of EZH2. a. ITPR2 gene was ablated in human T cells using CRISPR-Cas9 approach. **b.** Knockout efficiency was examined using Sanger Sequencing. **c.** Bar graphs show the transcription of IFNG and IL2 measured by qPCR in ITPR2-ablated human CD4$^+$ and CD8$^+$ T cells. (**d-f**) flow cytometry measurement of cytokine production by TCR-activated WT and I2KO T cells 7 days after

activation, with or without TAZ treatment for 3 days. **g.** Bar graphs show the transcription of ITPR2 in TAZ-treated human CD4$^+$ and CD8$^+$ T cells measured by qPCR. **h.** Bar graphs show the recovery rate of TAZ-treated TCR-activated CD4$^+$ T cells when ITPR2 was ablated. Data are presented as mean ± SD. Statistical analyses were performed as described in the Methods. *p < 0.05, **p <0.01, ***p <0.001, ****p < 0.0001.

Supplementary Fig.6. Evaluation of mouse hCD19-CAR-T Cell cytotoxicity. a. Expression of human CD19 on engineered C1498 acute myeloid leukemia (AML) cells. **b.** Formula used to calculate the cytotoxicity of hCD19-CAR-T cells against tumor cells. **c.** Cytotoxicity of WT versus EI2KO CAR-T cells against hCD19-C1498 AML cells. **d.** Representative flow plots of tumor cell coculture system. **e-f.** Comparison of IFN-γ levels between hCD19-CAR-T cells and non-CAR-T control cells. Representative data of 2-3 independent experiments were shown. Student's t-test was used for two-group comparison. ****p<0.0001.

Supplementary Fig.7. InsP3R2-mediated Ca^{2+} signals induce acute phase defects of Ezh2-deficient CAR T cells. Equal numbers (1x 10^6 cells) of WT, EKO and EI2KO CAR CD4$^+$ and CD8$^+$ T cells were transferred into hCD19-C1498 AML-bearing B6 mice. Spleen, LN, BM and liver tissues were harvested for CAR-T cells immunophenotyping and function assay. **a.** Graphs show the number of T_{EM}-phenotype CD8$^+$CAR-T cells in BM and Spleen. **b.** Representative flow plots of IFNg$^+$CD4$^+$ CAR-T cells in BM. **c.** Graphs show the percentage of T_{CM} (CD44$^+$CD62L$^+$) and T_{EM} (CD44$^+$CD62L$^-$) in CD8$^+$CAR-T cells in BM and Spleen. **d-f.** Plots and graphs show the percentage of IFN-γ$^+$ cells in CD8$^+$CAR-T cells in BM and Spleen and the expression level of IFN-γ. **g.** Graphs show the percentage of PD-1$^+$CD39$^+$ cells in CD8$^+$ CAR-T cells in Spleen, BM and Liver. One-way ANOVA followed by post-hoc tests was performed for pairwise comparisons among multiple groups. *p<0.05, **p<0.01, ***p<0.001, ****p<0.0001.

Supplementary Fig.8. a-b. Tacrolimus-pretreatment of murine CAR T cells enhances their EZH2 function and antitumor activity. hCD19-41BB-eGFP CAR T cells (CD4$^+$ and CD8$^+$) of B6 mouse origin were treated with tacrolimus (Tac, 50 ng/ml) or PBS from day 3 to day 6 after TCR activation. CAR T cells were harvested 6 days after culture. **a.** Western blots show the expression of EZH2 protein and H3K27me3 levels in CAR T cells treated with or without Tac for 3 days. **b.** qPCR analysis shows the mRNA levels in CAR T cells treated with or without Tac. **c-d**. CD8$^+$ CAR T cells were incubated in the presence or absence of BTP2 (1.0 μM) from day 3 to day 6 after TCR activation. Western blots show the expression of EZH2 and H3K27me3 (**c**). Real-time RT-PCR show the mRNA level of each gene (**d**). Representative data of 2-3 independent experiments were shown. Student's t-test was used for two-group comparison. *p<0.05, **p<0.01, ***p<0.001.

Supplementary Fig.9. Tac-pretreated CAR T cells exhibit enhanced antitumor activity. a. hCD19-41BB-eGFP CAR T cells (CD4$^+$ and CD8$^+$) of B6 mouse origin were treated with tacrolimus (Tac, 50 ng/ml) or PBS from day 3 to day 6 after TCR activation. CAR T cells were harvested 6 days after culture. Tac- or PBS-treated CAR T cells (4x10^5 CD4$^+$ CAR T cells and 4x10^5 CD8$^+$ T cells) were infused (i.v.) into B6 mice that had been inoculated with hCD19-C1498 cells 7 days earlier. **b.** Representative images of tumor luciferase activity detected with IVIS. **c.** Survival curves of leukemia-bearing B6 mice. **d.** Graphs show the percentage of PD-1$^+$CD39$^-$ progenitor-like Tex CAR CD4$^+$ and CD8$^+$ T cells isolated from the spleens and livers of recipient mice 9 days after CAR T cells infusion. **e**. Graphs show the percentage of

PD-1^{+}CD39^{+} term-like Tex CAR CD4^{+} and CD8^{+} T cells isolated from the spleens and livers of recipient mice 9 days after CAR T cells infusion. Statistical analysis of mice survival was performed with the Log-rank (Mantel–Cox) test. Representative data of 2 independent experiments were shown. Student's t-test was used for two-group comparison. *p<0.05, **p<0.01, ***p<0.001.

a

Upstream regulators for genes targeted by Ezh2 in activated CD8⁺ T cells

Upstream Regulator	Activation z-score	p-value of overlap
Ca^{2+}	1.949	4.40E-06
Ionomycin	2.58	6.68E-05
NFAT(family)	2.2	5.45E-05
thapsigargin	2.613	2.36E-04
IL2	3.135	4.00E-19
Cyclosporin A	-2.49	1.33E-11
Tacrolimus	-2.467	5.10E-11

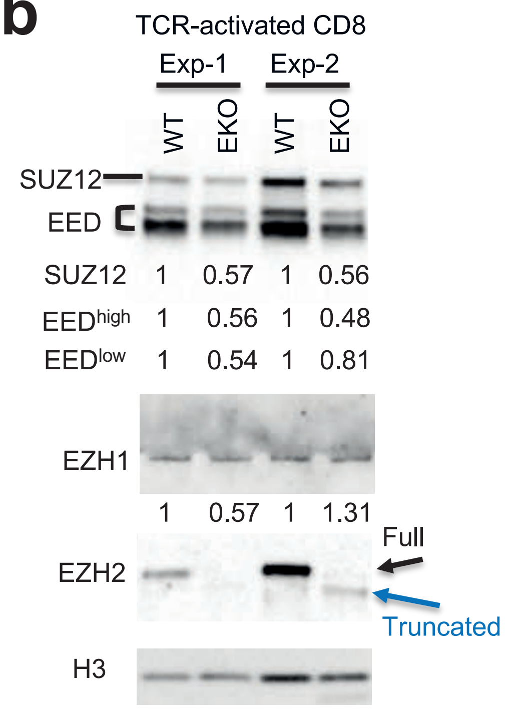

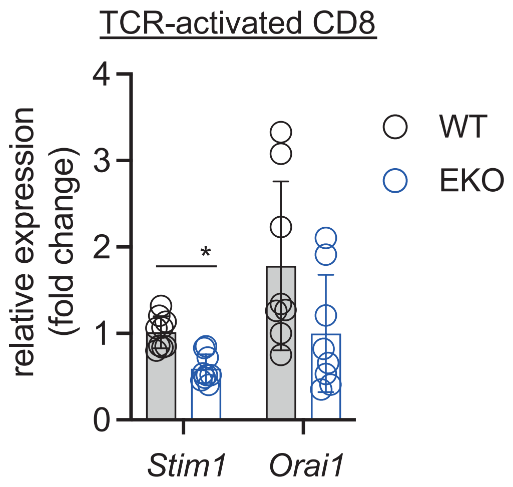

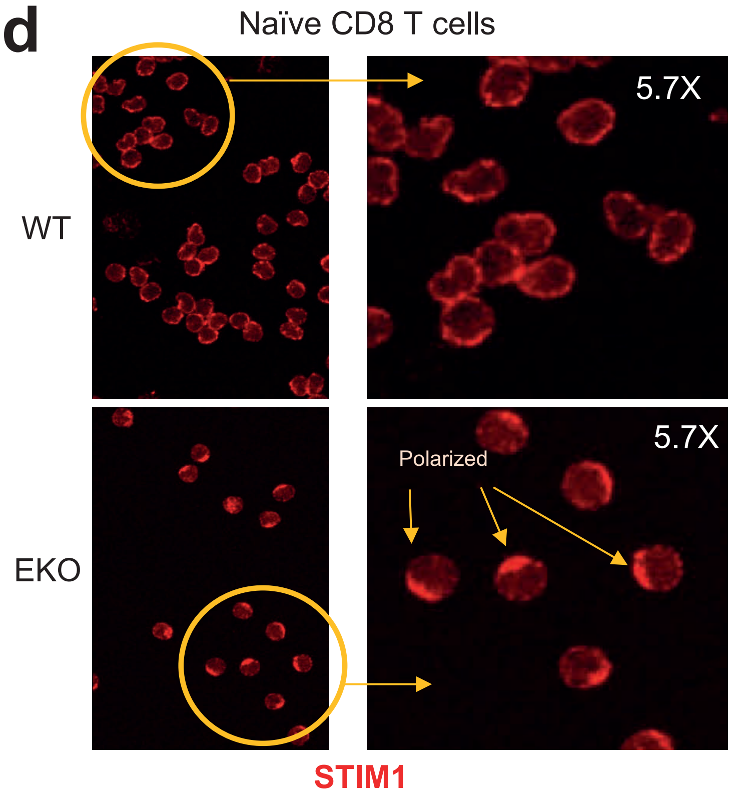

Fig.s1

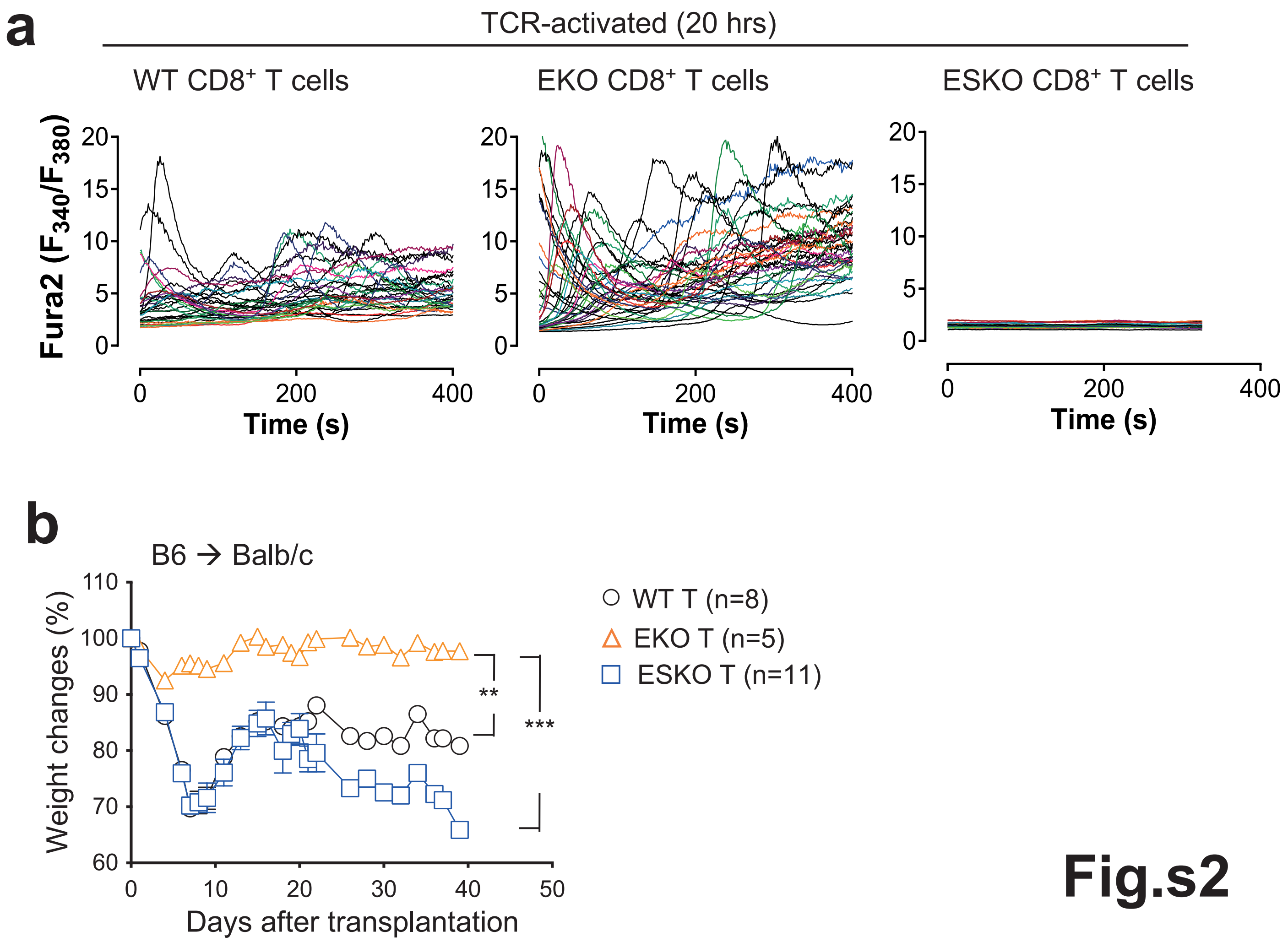
a
TCR-activated (20 hrs)
WT CD8+ T cells
EKO CD8+ T cells
ESKO CD8+ T cells
Fura2 (F340/F380)
20
15
10
5
0
0
200
400
Time (s)
b
B6 → Balb/c
Weight changes (%)
110
100
90
80
70
60
0
10
20
30
40
50
Days after transplantation
WT T (n=8)
EKO T (n=5)
ESKO T (n=11)
**
***

Fig.s2

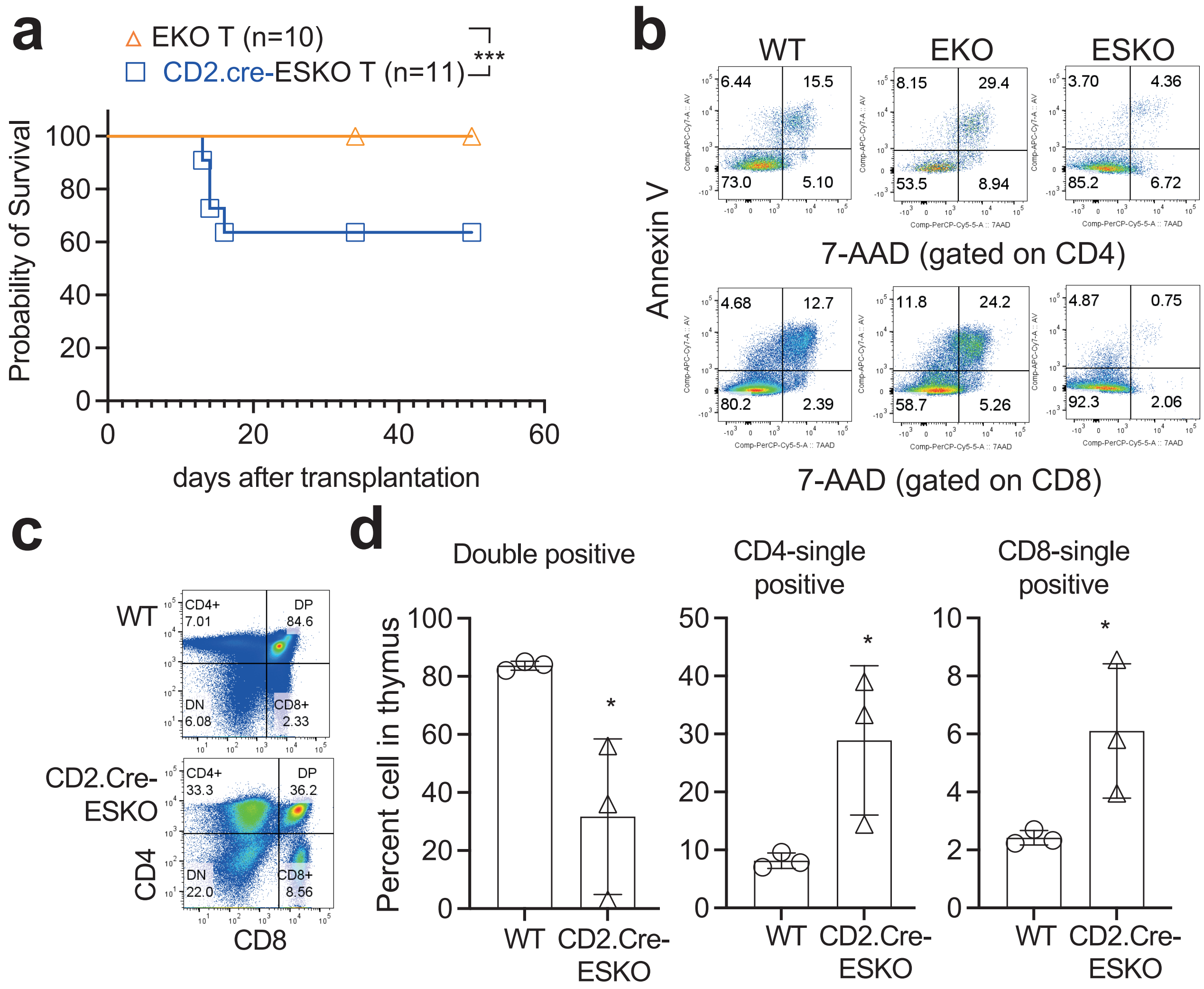
a
EKO T (n=10)
CD2.cre-ESKO T (n=11)
***
Probability of Survival
100
80
60
40
20
0
0
20
40
60
days after transplantation
b
WT
EKO
ESKO
Annexin V
6.44
15.5
73.0
5.10
8.15
29.4
53.5
8.94
3.70
4.36
85.2
6.72
7-AAD (gated on CD4)
4.68
12.7
80.2
2.39
11.8
24.2
58.7
5.26
4.87
0.75
92.3
2.06
7-AAD (gated on CD8)
c
WT
CD4+
7.01
DP
84.6
DN
6.08
CD8+
2.33
CD2.Cre-
ESKO
CD4+
33.3
DP
36.2
DN
22.0
CD8+
8.56
CD4
CD8
d
Double positive
CD4-single positive
CD8-single positive
Percent cell in thymus
100
80
60
40
20
0
50
40
30
20
10
0
10
8
6
4
2
0
*
*
*
WT
CD2.Cre-ESKO
WT
CD2.Cre-ESKO
WT
CD2.Cre-ESKO

Fig.s3

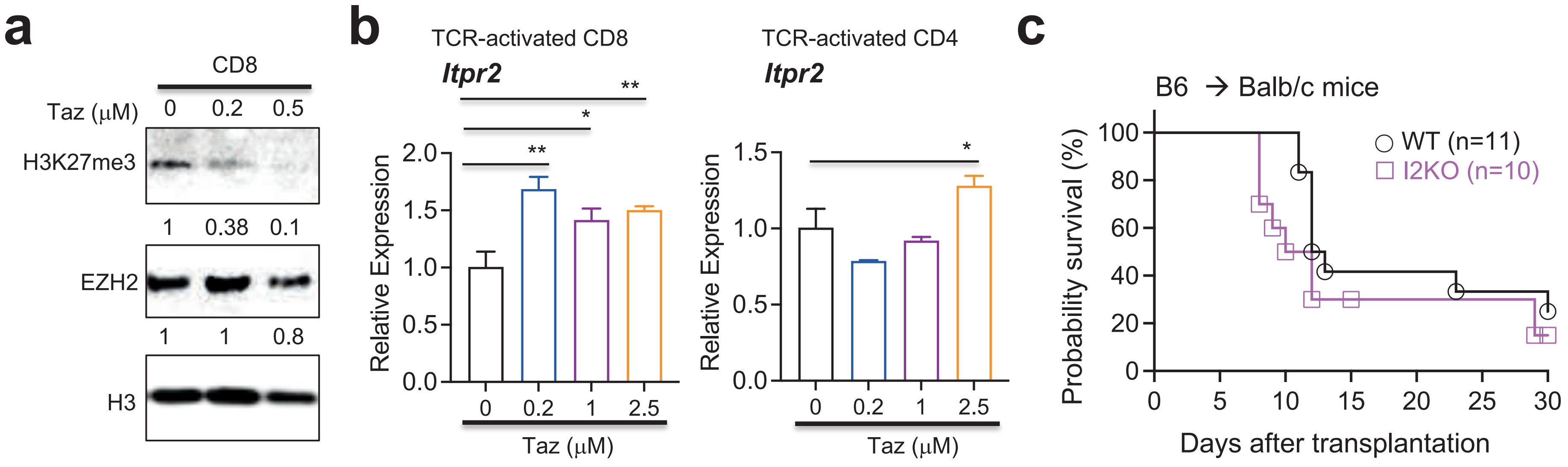

a
CD8
Taz (μM)
0
0.2
0.5
H3K27me3
1
0.38
0.1
EZH2
1
1
0.8
H3
b
TCR-activated CD8
Itpr2
Relative Expression
2.0
1.5
1.0
0.5
0.0
**
*
**
0
0.2
1
2.5
Taz (μM)
TCR-activated CD4
Itpr2
Relative Expression
1.5
1.0
0.5
0.0
*
0
0.2
1
2.5
Taz (μM)
c
B6 → Balb/c mice
Probability survival (%)
100
80
60
40
20
0
WT (n=11)
I2KO (n=10)
0
5
10
15
20
25
30
Days after transplantation

Fig.s4

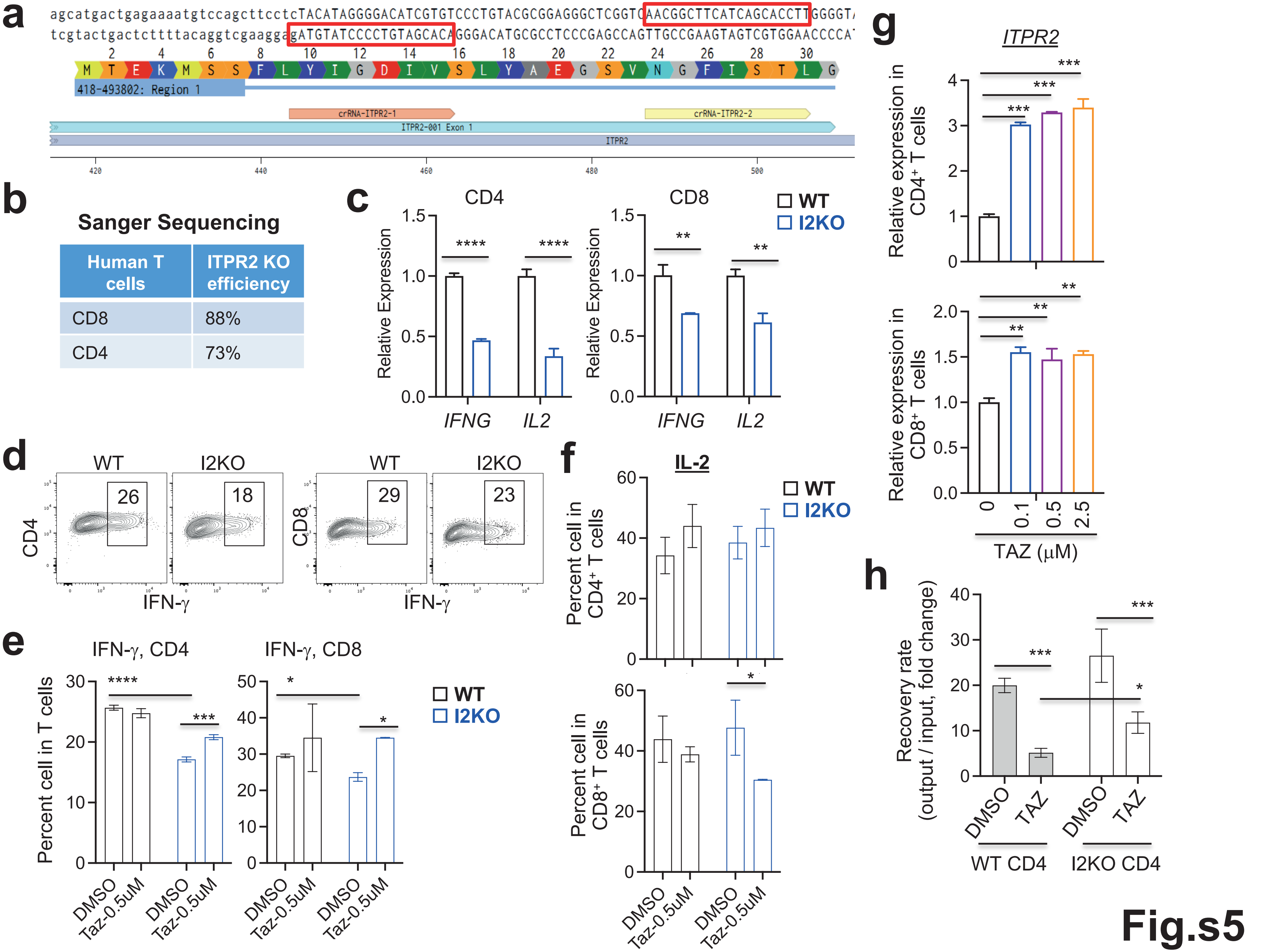
a
agcatgactgagaaaatgtccagcttcctcTACATAGGGGACATCGTGTCCCTGTACGCGGAGGGCTCGGTCAACGGCTTCATCAGCACCTTGGGGT
tcgtactgactcttttacaggtcgaaggaGATGTATCCCCTGTAGCACAGGGACATGCGCCTCCCGAGCCAGTTGCCGAAGTAGTCGTGGAACCCCA
M T E K M S S F L Y I G D I V S L Y A E G S V N G F I S T L G
418-493802: Region 1
crRNA-ITPR2-1
crRNA-ITPR2-2
ITPR2-001 Exon 1
ITPR2
420 440 460 480 500
b
Sanger Sequencing
Human T cells | ITPR2 KO efficiency
CD8 | 88%
CD4 | 73%
c
CD4
CD8
WT
I2KO
Relative Expression
IFNG
IL2
****
**
d
WT
I2KO
26
18
29
23
CD4
CD8
IFN-γ
e
IFN-γ, CD4
IFN-γ, CD8
Percent cell in T cells
DMSO
Taz-0.5uM
f
IL-2
Percent cell in CD4+ T cells
Percent cell in CD8+ T cells
g
ITPR2
Relative expression in CD4+ T cells
Relative expression in CD8+ T cells
TAZ (μM)
0
0.1
0.5
2.5
h
Recovery rate (output / input, fold change)
DMSO
TAZ
WT CD4
I2KO CD4

Fig.s5

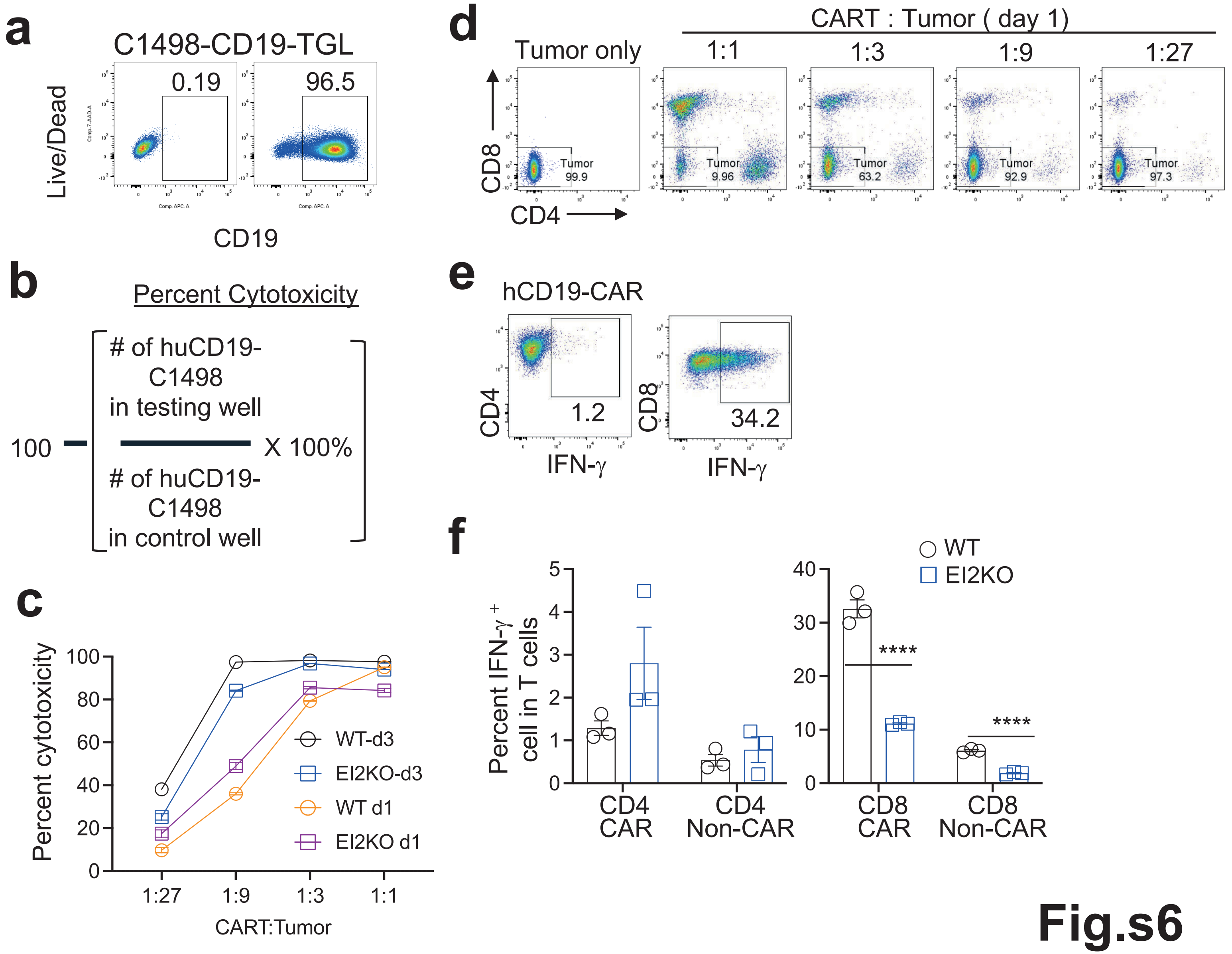
a
C1498-CD19-TGL
Live/Dead
CD19
0.19
96.5
b
Percent Cytotoxicity
100 − [# of huCD19-C1498 in testing well / # of huCD19-C1498 in control well X 100%]
c
Percent cytotoxicity
CART:Tumor
1:27
1:9
1:3
1:1
WT-d3
EI2KO-d3
WT d1
EI2KO d1
d
CART : Tumor (day 1)
Tumor only
1:1
1:3
1:9
1:27
CD8
CD4
Tumor 99.9
Tumor 9.96
Tumor 63.2
Tumor 92.9
Tumor 97.3
e
hCD19-CAR
CD4
CD8
IFN-γ
1.2
34.2
f
Percent IFN-γ+ cell in T cells
WT
EI2KO
CD4 CAR
CD4 Non-CAR
CD8 CAR
CD8 Non-CAR
****
****

Fig.s6

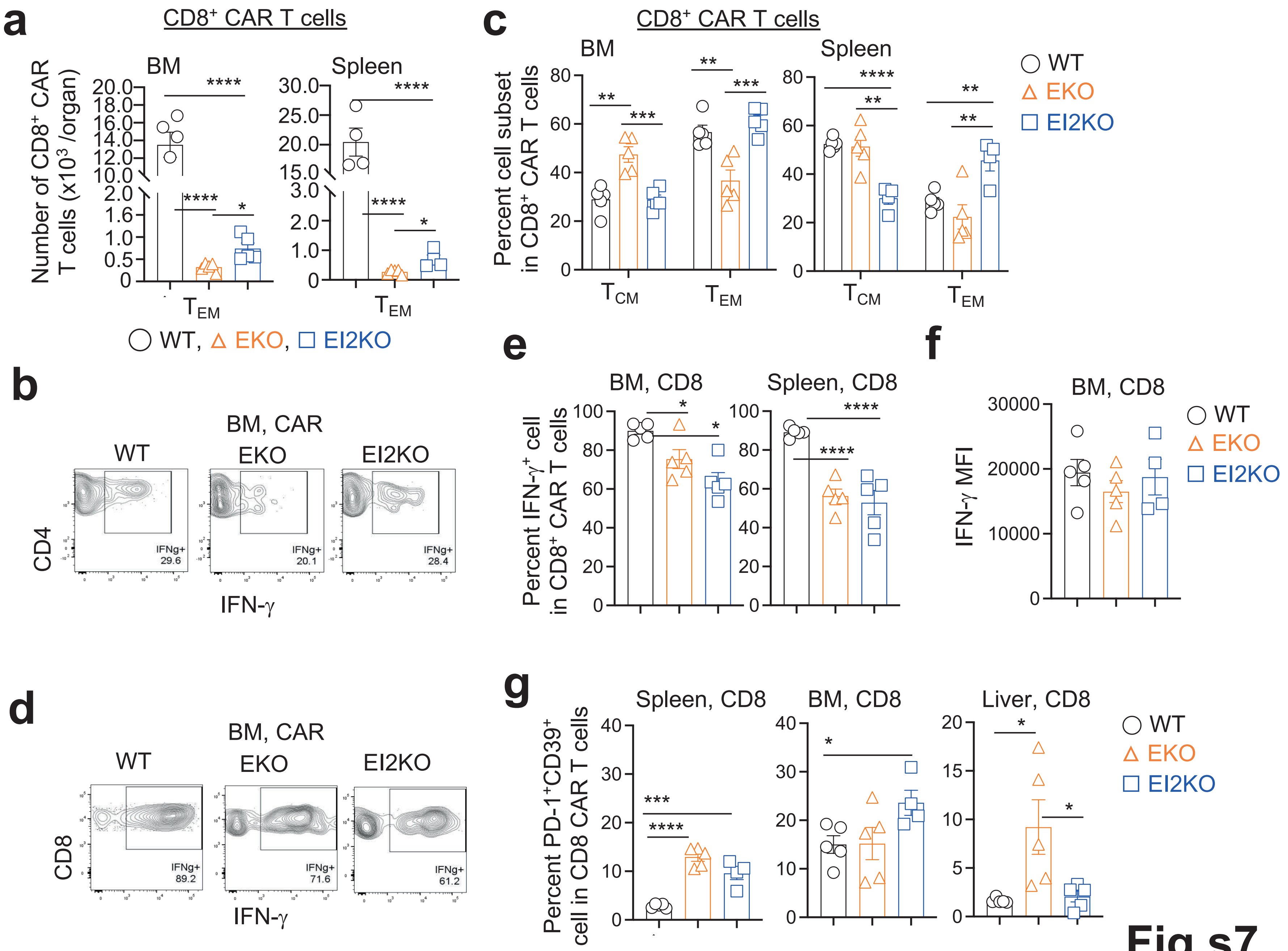

a
CD8+ CAR T cells
BM
Spleen
Number of CD8+ CAR T cells (x10³ /organ)
TEM
WT, EKO, EI2KO
b
BM, CAR
WT
EKO
EI2KO
CD4
IFN-γ
IFNg+ 29.6
IFNg+ 20.1
IFNg+ 28.4
c
CD8+ CAR T cells
BM
Spleen
Percent cell subset in CD8+ CAR T cells
TCM
TEM
WT
EKO
EI2KO
d
BM, CAR
WT
EKO
EI2KO
CD8
IFN-γ
IFNg+ 89.2
IFNg+ 71.6
IFNg+ 61.2
e
BM, CD8
Spleen, CD8
Percent IFN-γ+ cell in CD8+ CAR T cells
f
BM, CD8
IFN-γ MFI
WT
EKO
EI2KO
g
Spleen, CD8
BM, CD8
Liver, CD8
Percent PD-1+CD39+ cell in CD8 CAR T cells
WT
EKO
EI2KO

Fig.s7

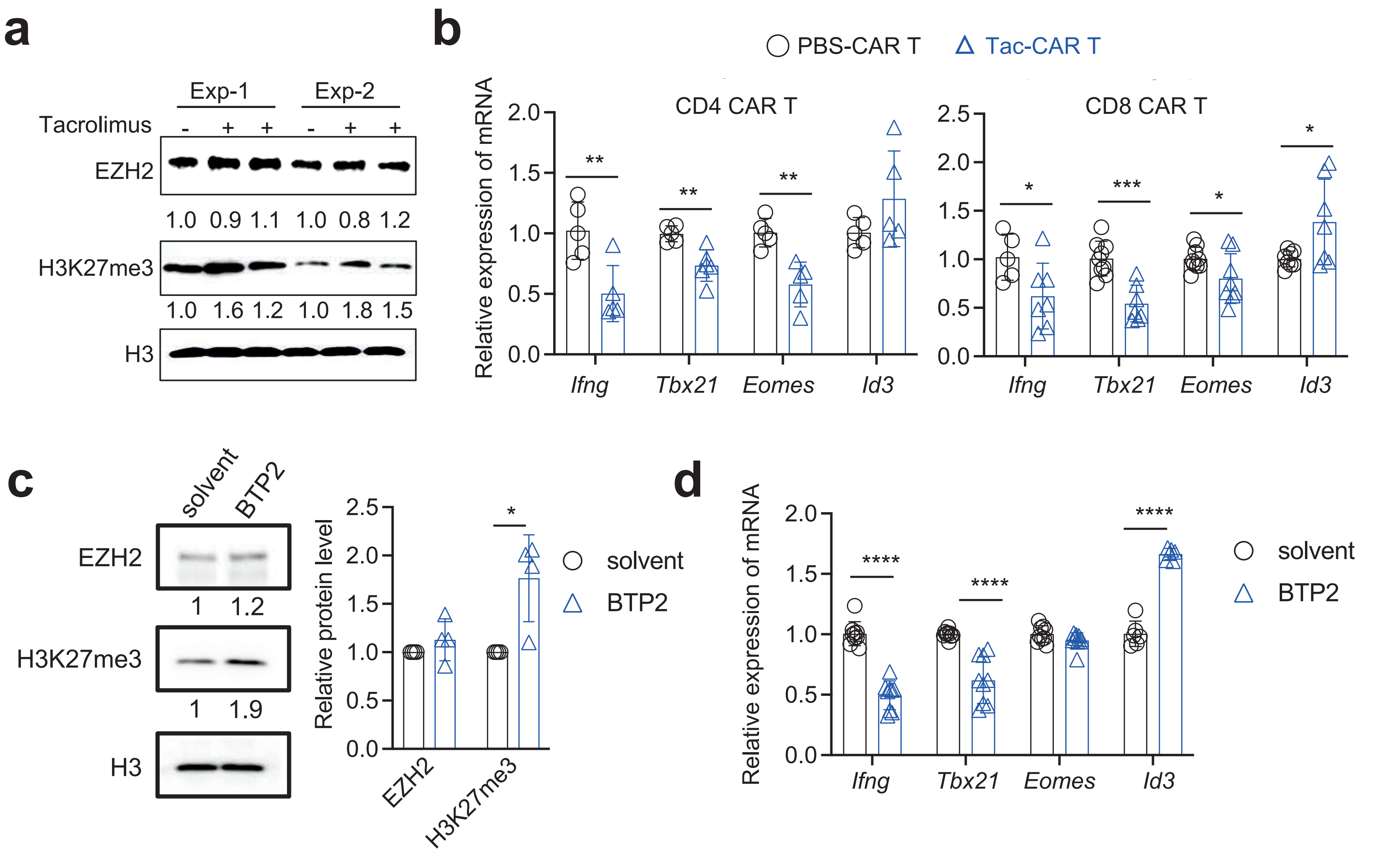
a
Exp-1
Exp-2
Tacrolimus
- + + - + +
EZH2
1.0 0.9 1.1 1.0 0.8 1.2
H3K27me3
1.0 1.6 1.2 1.0 1.8 1.5
H3
b
PBS-CAR T
Tac-CAR T
CD4 CAR T
CD8 CAR T
Relative expression of mRNA
Ifng
Tbx21
Eomes
Id3
c
solvent
BTP2
EZH2
1 1.2
H3K27me3
1 1.9
H3
Relative protein level
d
Relative expression of mRNA

Fig.s8

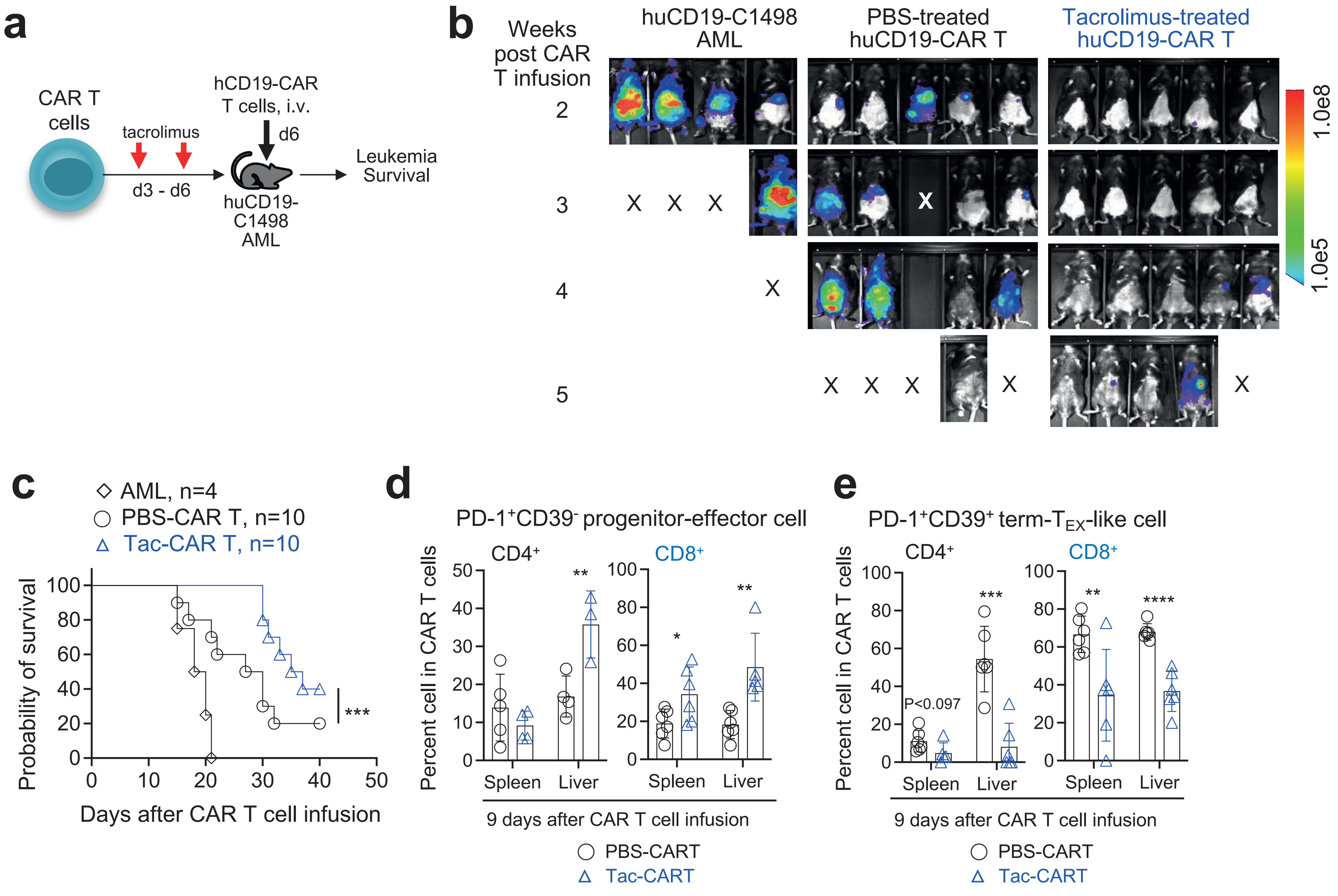
a
CAR T cells
tacrolimus
d3 - d6
hCD19-CAR T cells, i.v.
d6
huCD19-C1498 AML
Leukemia Survival
b
Weeks post CAR T infusion
huCD19-C1498 AML
PBS-treated huCD19-CAR T
Tacrolimus-treated huCD19-CAR T
2
3
4
5
1.0e8
1.0e5
c
AML, n=4
PBS-CAR T, n=10
Tac-CAR T, n=10
Probability of survival
Days after CAR T cell infusion
***
d
PD-1+CD39- progenitor-effector cell
CD4+
CD8+
Percent cell in CAR T cells
Spleen
Liver
9 days after CAR T cell infusion
PBS-CART
Tac-CART
e
PD-1+CD39+ term-TEX-like cell
CD4+
CD8+
P<0.097
Percent cell in CAR T cells
Spleen
Liver
9 days after CAR T cell infusion
PBS-CART
Tac-CART

Fig.s9
