# Supplementary figures and images for: EZH2 and intracellular Ca2+ signals interdependently coordinate alloreactive and CAR-T-cell responses
Source: Cell Mol Immunol. 2026 Apr 22;23(7):840–54. doi: 10.1038/s41423-026-01413-y (PMC13314966; doi:10.1038/s41423-026-01413-y)

**Fig.2****a**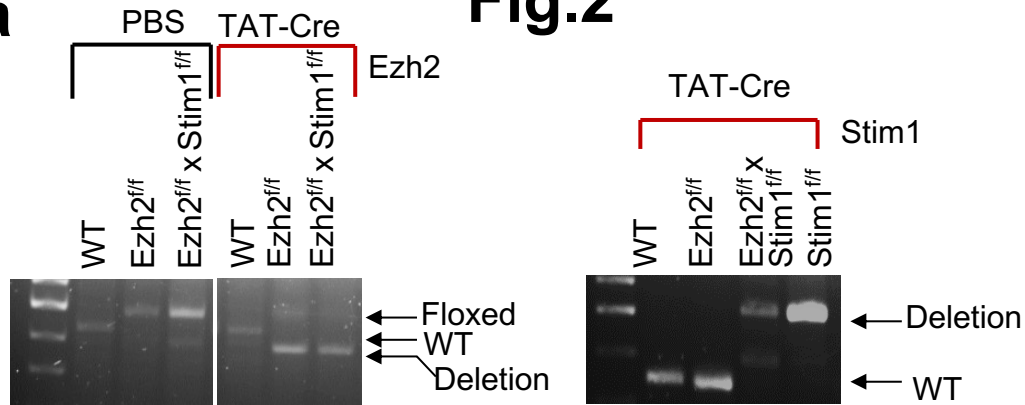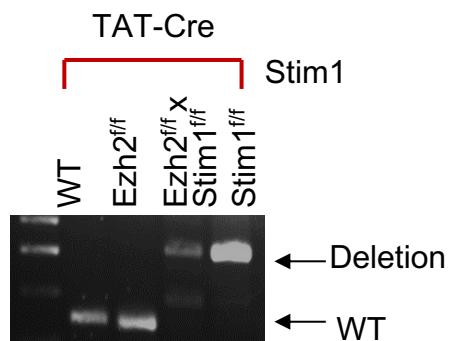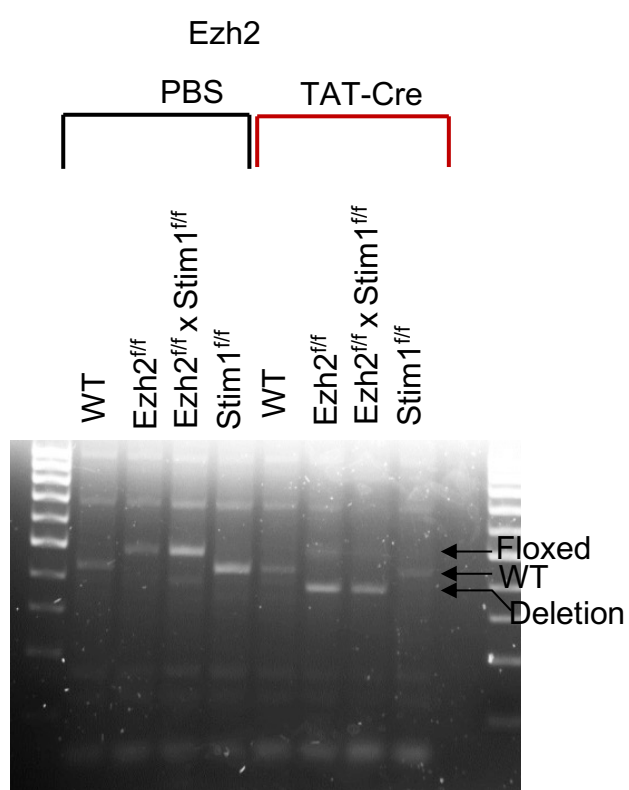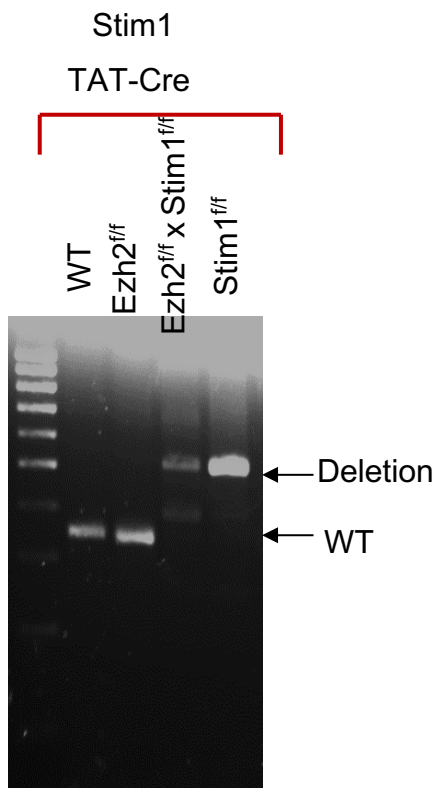

# Fig.s1

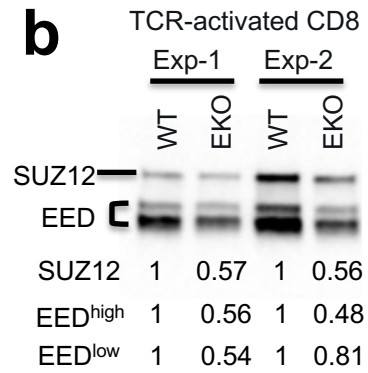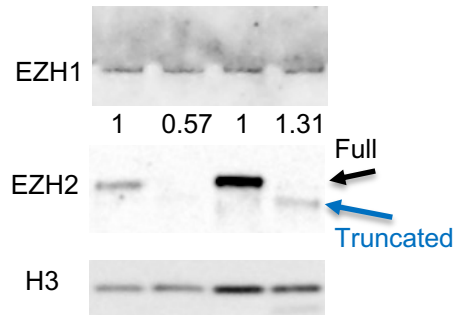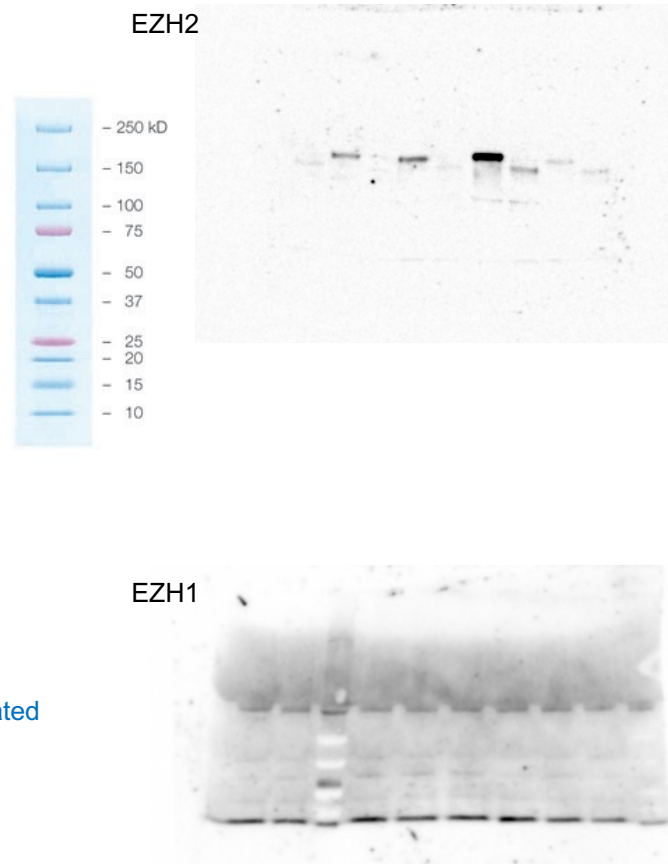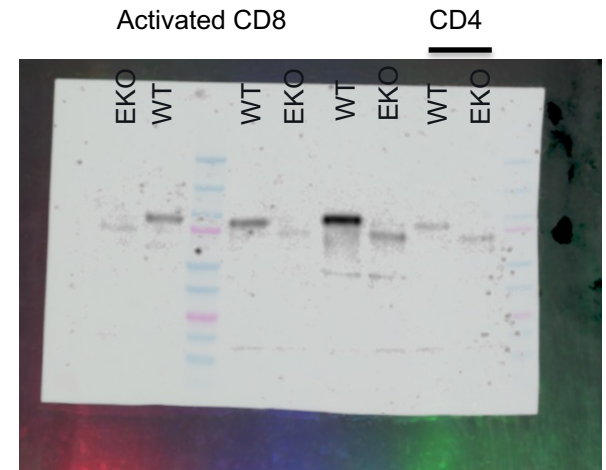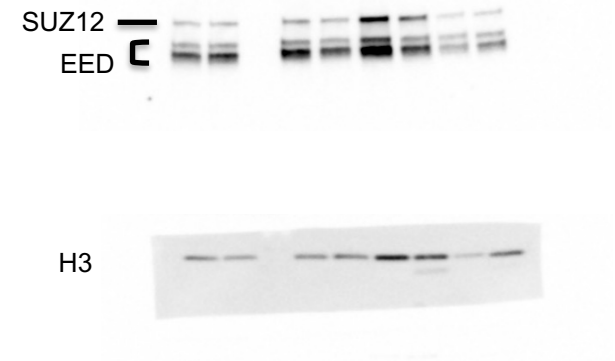

**Fig.s4**

**a**

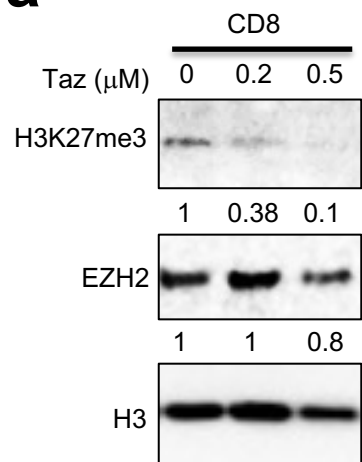

EZH2

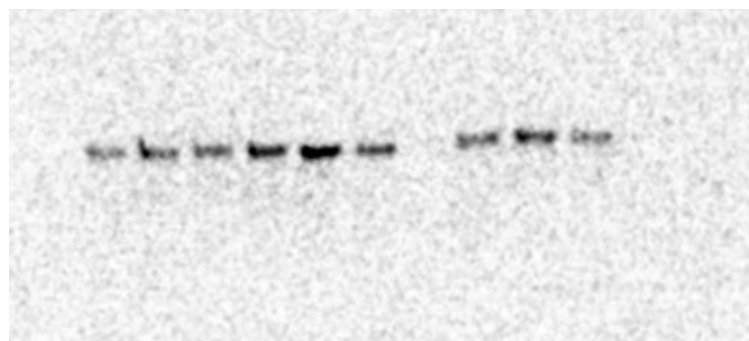

H3K27me3

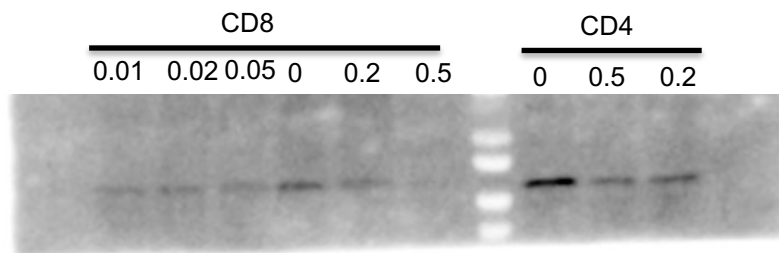

H3

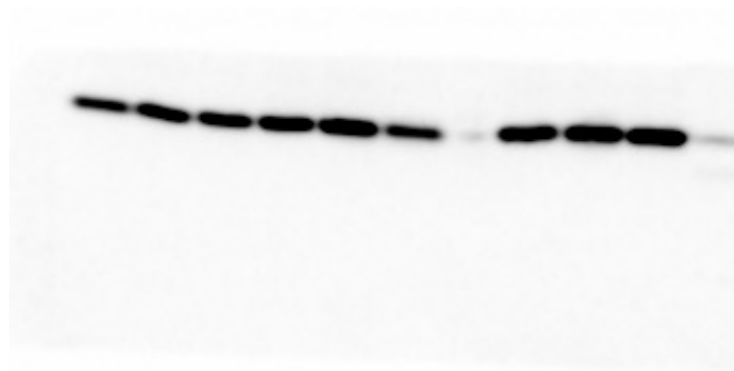

# Fig.s8

**a**

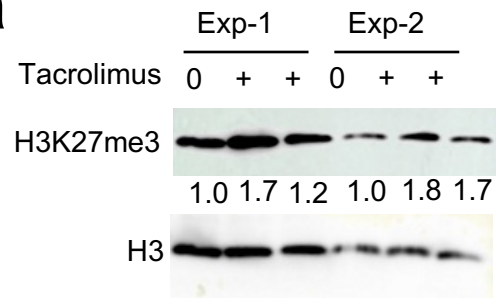

H3K27me3

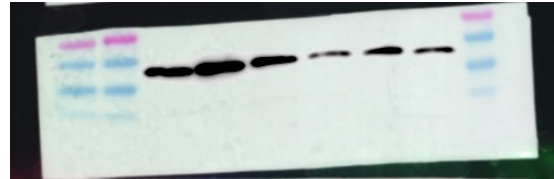

H3

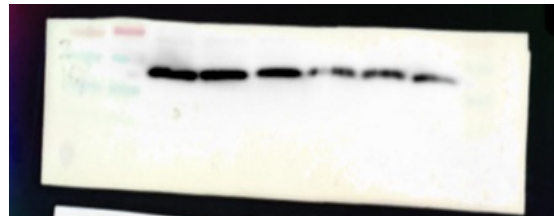

**c**

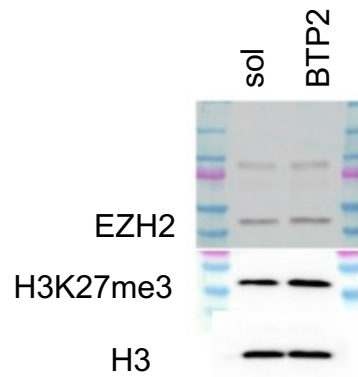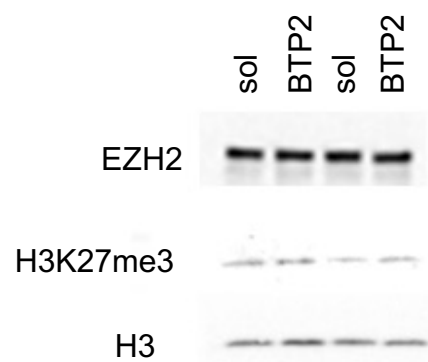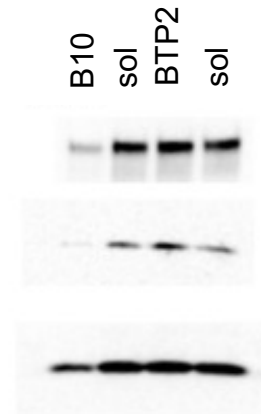

Supplement: Supplementary file 4 — unprocessed images [file 41423_2026_1413_MOESM4_ESM.pdf]
